# Barriers and facilitators to participation in physical activity for students with disabilities in an integrated school setting: a meta-synthesis of qualitative research evidence

**DOI:** 10.3389/fpubh.2025.1496631

**Published:** 2025-03-12

**Authors:** Xinyi Liu, Haijun Han, Zhendong Li, Shouzhen Huang, Yijia Zhao, Qian Xiao, Jingquan Sun

**Affiliations:** ^1^Institute of Sports Science, Sichuan University, Chengdu, China; ^2^School of Physical Education, Sichuan University, Chengdu, China; ^3^Institute of Art College, Sichuan University, Chengdu, China

**Keywords:** students with disabilities, physical activity, integrated school, facilitators, barriers, systematic review

## Abstract

**Purpose:**

This review investigates the factors influencing the participation of students with disabilities (SWD) in physical activities (PA) within integrated school settings.

**Materials and methods:**

A keyword search of articles published up to May 2024 identified 22 studies meeting the inclusion criteria. Thematic synthesis was used to analyze the data, resulting in a dynamic theoretical model.

**Results:**

The model encompasses 19 themes, including an individual-level “Special factor” and three phases: perspectives from school administrators (First phase), a two-way dialogue between school management and staff (Second phase), and interactions among staff, SWD, and peers (Third phase). The final phase, based on basic psychological needs theory, identifies autonomy, competence, and relatedness needs.

**Conclusion:**

The dynamic model highlights that no single factor fully explains SWD participation in PA within inclusive environments. Educators must consider physiological, behavioral, and cognitive aspects, as well as mediating factors and processes (three phases), to design tailored strategies that address SWD needs and foster a supportive environment.

**Systematic review registration:**

https://www.crd.york.ac.uk/PROSPERO/view/CRD42024577620.

## 1 Introduction

The concept of inclusive physical education (PE) is widely practiced internationally. Inclusive PE aims to create an environment where students with disabilities (SWD) and those without disabilities learn together, addressing the needs of SWD as a marginalized group (1, 2). Inclusive education has seemingly secured the right to quality education for all students (3). Over the past 30 years, integrated school placements and the philosophy of inclusion have become prominent trends in global education (4–6). For example, education laws in many countries, such as Brazil and Japan, now mandate that SWD be educated in integrated settings wherever possible (7). In the United States, up to 96% of SWD are educated in integrated settings, while only 3% of SWD in European countries attend separate institutions outside the compulsory education system (8–12).

Conceptually, integrated education offers undeniable benefits, such as increased social interaction, a sense of belonging, and the development of sports skills and experiences during adolescence and young adulthood, which can lay the foundation for lifelong behavioral patterns (13, 14). However, the practical implementation of integrated education has been criticized by scholars for often forcing SWD to conform to traditional and normative educational processes, thereby perpetuating segregation (15, 16). A significant gap in the current literature is the limited focus on how rigid PE curricula and the failure to adapt them to the needs of SWD further entrench segregation. While many studies highlight the benefits of inclusive education, they often neglect the structural barriers that prevent meaningful participation of SWD in mainstream educational practices. Additionally, it has been found that school administrators may manipulate student placements based on financial or scheduling constraints (17). Consequently, SWD often experience limited success in learning and infrequent social interactions with non-disabled peers in inclusive settings, which contradicts the optimistic claims made in the literature (5, 18, 19). This underscores the urgent need for research to address how institutional and structural factors interact with the lived experiences of SWD, which remains underexplored.

Generally, individuals with disabilities are less likely to participate in regular physical activities (PA) compared to their non-disabled counterparts. However, they have the same needs for promoting health, preventing disease, and reducing secondary conditions associated with disabilities. This is particularly critical during the transition from adolescence to young adulthood, a key period for establishing long-term physical activity habits and addressing risk factors for chronic health conditions (20). PA can also enhance psychosocial well-being by fostering a sense of belonging, reducing social isolation, and improving quality of life (21). However, the actual participation rates of SWD in PA within integrated schools remain low, and they often fail to meet recommended physical activity guidelines (22). This gap highlights the significant barriers SWD face in accessing physical activity opportunities. In 2008, the U.S. Congress mandated the Government Accountability Office to investigate how schools provide opportunities for SWD to engage in PE and extracurricular activities. The study revealed numerous barriers to integrating SWD into general PE programs, resulting in limited opportunities and unequal access compared to their peers (23). PE is regarded as one of the first school-based curricula and serves as the primary channel for SWD to engage in PA within the school setting (24). However, school PE classes have strayed from their original goal of providing equitable education to all students (25). A lack of formal instructional strategies and guidance has left PE professionals uncertain about how to effectively address the needs of SWD and promote social and physical inclusion in PE classes (6). As a result, inadequate participation in PE has led to SWD being legally excluded from the same educational opportunities as their peers (26).

There has been a growing body of research examining the factors that influence PA participation among adolescents/students with disabilities. The research subjects have also diversified, including studies focused on specific disability types, onset during childhood (27), and disabled adolescents/students who are athletes (28). Additionally, some studies investigate the attitudes of SWD parents (29), PE teachers, Adaptive physical education teachers (APE), trainee teachers, and peers towards students with special educational needs (4, 30), as well as the behaviors of disabled and non-disabled students in integrated PE. The settings for these studies are also diverse, ranging from examining barriers to PA participation in gyms (31) to PE settings (32). However, despite the increasing trend of SWD participating in regular PE classes in integrated school environments, much of the existing research tends to focus on PA participation in external environments or isolated PE contexts (33, 34). This narrow approach overlooks the broader reality that SWD are not isolated in these environments. There is a critical gap in research regarding the complex and interrelated factors that influence PA participation across the entire integrated school setting, including interactions with peers, teacher attitudes, school policies, and extracurricular opportunities. This gap limits our understanding of how the full school context can either support or hinder PA participation for SWD.

Studies examining the factors influencing SWD participation in PA often rely on qualitative methods, which provide valuable insights into their experiences. However, there is a noticeable lack of comprehensive reviews that synthesize multiple perspectives and experiences within integrated PE environment, While narrative reviews are common, they often lack systematic search strategies and objective conclusions, and their subjective inclusion criteria and data extraction methods can introduce bias (35). Furthermore, systematic reviews often draw on behavioral epidemiology frameworks and socio-ecological models (36), which, although effective in public health research, may not fully capture the nuances of educational contexts. These models can introduce biases when interpreting qualitative findings on student behavior and provide limited guidance on practical implementation in school settings, thereby restricting their applicability in addressing systemic educational challenges.

A new theoretical framework tailored to the educational context is essential. The Basic Psychological Needs Theory (BPN), a core component of Self-Determination Theory (SDT), offers a promising alternative. BPN identifies three fundamental psychological needs—autonomy, competence, and relatedness—as essential for individuals’ well-being and psychological growth. Motivation plays a key role in influencing participation in physical activity, as fulfilling these basic needs can enhance intrinsic motivation, leading to more sustained engagement in PA. As noted by Ahmadi (37), distinguishing between intrinsic and extrinsic forms of motivation is crucial, as they are differently related to positive and negative outcomes. This framework has been widely applied in education, healthcare, and sports (38–41), and its focus on the satisfaction or frustration of these needs provides a robust basis for understanding motivation and behavior. In PE, BPNT helps explain how conditions influence students’ interest in physical activity. By addressing these psychological needs, educators can create inclusive and supportive environments that align systemic factors—such as school culture, teacher training, and peer relationships—with SWD’s psychological well-being and participation in PA. This holistic approach is crucial as the increasing number of SWD in integrated educational environments highlights the need for practical and evidence-based strategies to promote their engagement in physical activity”.

In summary, given the increasing number of SWD entering integrated educational environments underscores the importance of understanding the factors that either facilitate or hinder their participation in PA. Despite this, existing research fails to provide systematic reviews or practical guidance to effectively promote PA in these settings. Many reviews focus narrowly on specific contexts, such as PE classes or extracurricular activities, without considering the broader and more complex dynamics of integrated school environments. This fragmented approach limits a holistic understanding of how systemic factors, such as school culture, teacher training, and peer relationships, influence SWD’s participation in PA.

Additionally, prior research predominantly adopts a “one-way” dialogue approach, focusing on a single group of stakeholders, such as students or teachers, while neglecting the interconnected perspectives of others. This method overlooks the opportunity to generate richer insights through cross-pollination of ideas among students, parents, teachers, and school administrators. Consequently, important interconnections between factors affecting SWD’s PA participation remain unexplored.

To address these limitations, this study adopts a “multi-way” dialogue model, incorporating the perspectives of SWD, parents, teachers, and other key stakeholders. By exploring how these diverse viewpoints interact to shape the barriers and facilitators of PA participation, this research seeks to provide a more comprehensive understanding of the dynamics at play. This holistic approach enables a nuanced interpretation of the factors influencing SWD’s engagement in PA, moving beyond the one-dimensional focus of previous studies. Ultimately, this study aims to contribute to the development of more effective educational practices and policies that promote inclusive PA participation in integrated school settings.

### 1.1 Review questions

Based on the identified gaps in current literature, this study aims to address the following key research questions:

- What are the specific barriers and facilitators that SWD face in participating in PA within inclusive school settings, and how are these factors interrelated?
- How can various stakeholders—such as students, parents, teachers, and administrators—use these influencing factors to enhance their educational practices and arrangements for SWD to promote PA participation?

## 2 Method

The structure of this systematic review adheres to the PRISMA (Preferred Reporting Items for Systematic Reviews and Meta-Analyses) guidelines (42). This study has been pre-registered with PROSPERO (Registration number: CRD42024577620).

### 2.1 Search strategy

Due to the unique structure of each database, accessing and searching these resources require the use of the same terms with different strategies. Two researchers (XL, SH) conducted a comprehensive search of peer-reviewed articles and theses published up to May 2024 in the Web of Science, EBSCO (APA, CINAHI, ERIC), PubMed, and Scopus databases, excluding the Preprint Citation Index (see Supplementary Data A). The search strategy used various combinations of three types of terms: (a) population (e.g., middle school, high school, college students, other stakeholders); (b) population characteristics (“mobility impairment,” “sensory impairment”); (c) phenomena of interest (“attitudes,” “barriers,” “facilitators”); (d) physical activity (“exercise,” “sports”) and related synonyms. Additional relevant studies were identified by manually checking the reference lists of included studies and tracking citations using Google Scholar. Our search was limited to English-language articles. The purpose of our paper is to identify the factors influencing the participation of SWD in PA within integrated school environments. Since some studies may involve interviewing adults with disabilities who recall their school experiences or stakeholders (e.g., PE teachers, adaptive educators) discussing their views and attitudes towards SWD participation in PA, age was not restricted in the search strategy. However, to minimize errors due to significant age differences, the experiences in question were limited to integrated school contexts at the middle school, high school, and college levels, excluding primary schools and kindergartens. Therefore, age restrictions for SWD were part of the inclusion criteria (see “Inclusion Criteria” below).

### 2.2 Inclusion and exclusion criteria

#### 2.2.1 Inclusion criteria

##### 2.2.1.1 Context

SWD can be educated in two settings: special schools tailored for students with special educational needs or integrated schools where they learn alongside non-disabled students. This study focuses on factors affecting SWD participation in PA within integrated school settings; thus, only studies conducted in integrated educational settings were included. While there are conceptual differences among mainstream education environments, integrated school settings, and inclusive educational contexts, all three were included in this review. These environments share the characteristic of enabling students with disabilities to learn alongside their non-disabled peers, which aligns with the study’s focus on identifying factors influencing SWD participation in physical activity within such settings. Therefore, no strict distinctions were made between these terms in this review.

In addition, studies conducted in kindergarten and primary schools settings were not included because the ultimate goal of this review is to apply these factors to actual educational settings. These levels of education differ significantly from middle school, high school, and college in terms of pedagogical philosophy, developmental goals, and impact on students and stakeholders. This difference makes the findings of the lower age groups less applicable to the practical implications of this study. Therefore, only comprehensive educational contexts occurring in middle school, high school, and college were included in this review.

##### 2.2.1.2 Phenomena of interest

The phenomena of interest for this review are the perceived barriers and facilitators to SWD participation in physical activity, exercise, or recreational activities within integrated school settings. Barriers are defined as elements or phenomena that prevent something from occurring, while facilitators are elements that make something easier or help achieve it. Barriers and facilitators do not necessarily represent different phenomena; for instance, a deficiency or negative attribute can be considered a barrier, while a sufficient or positive attribute can be considered a facilitator. Studies that explore experiences related to these phenomena were included.

Notably, even if the factors influencing PA participation within the integrated school setting were only addressed as a subtopic or subcontext, the study was included if the reported subjects and research context met the inclusion criteria. This inclusion was determined after thorough discussion and agreement between the two researchers to ensure that studies aligned with the focus of the review.

##### 2.2.1.3 Participants

Adolescence is a transitional period marked by significant mental changes. To minimize errors due to large age differences, the included age range for students was set at 12–30 years (±2 years), covering middle school, high school, and college stages. However, to comprehensively capture relevant information, this review also considered studies involving other school stakeholders (e.g., teachers, parents, administrators) whose input may provide valuable insights. These stakeholders were not restricted by age, profession, or role. Instead, researcher (XL) screened whether their descriptions of experiences with physical activity participation aligned with the *Phenomena of interest* and *Context* of this review. This approach ensures that the study includes a wide range of perspectives while maintaining relevance to the research objectives.

##### 2.2.1.4 Types of studies

This study included English-language articles using qualitative data collection and analysis related to the phenomena of interest. Research designs considered were qualitative description, phenomenology, grounded theory, ethnography, action research, and mixed methods research.

#### 2.2.2 Exclusion criteria

The following studies were excluded: (1) those involving individuals with disabilities related to recent organ transplants or similar conditions; (2) studies focusing on chronic, transient, or unstable diseases (e.g., cancer, heart disease); (3) research not primarily conducted in inclusive educational settings (e.g., special schools, community, home environments); (4) studies set in kindergartens or elementary schools; (5) studies not examining barriers, facilitators, or attitudes toward participation in physical activity; (6) physical activities unrelated to the school environment, such as shopping, dog walking, or cooking; (7) studies focusing on the biomechanics (kinetics, kinematics, wheelchair propulsion), physiology (energy expenditure, muscle strength, metabolism), surgical treatments, therapeutic methods, orthopedic examinations, diagnostic techniques, or training programs related to disabilities; (8) reviews, interviews, letters, posters, book chapters, and books were also excluded. (9) Quantitative studies, including those presented narrative data derived from questionnaires.

### 2.3 Assessment of methodological quality

Two reviewers (XL, ZL) independently assessed the methodological quality of eligible studies. They compared and resolved differences through discussion and consensus, focusing on the validity, content, and applicability of the study results. The Critical Appraisal Skills Programme (CASP) Qualitative Checklist (43) was used to facilitate systematic evaluation. The CASP checklist was used to guide this process, helping ensure a consistent evaluation of the study’s overall quality. The responses were categorized as “yes” (✓), “cannot tell” (?), or “no” (✗).

To ensure consistency, the reviewers conducted a pilot assessment of three randomly selected studies to align their understanding of the checklist criteria. Discrepancies in ratings were resolved through discussion and consensus, and a third reviewer (SH) was available to mediate if agreement could not be reached. This process ensured reliability in the evaluation. Furthermore, the reviewers focused not only on whether the studies met individual checklist criteria but also on the overall quality and applicability of the results to the study’s objectives. Each study was given an overall quality rating based on its strengths and weaknesses in meeting the CASP criteria. The quality assessment results are provided in Supplementary Data B.

### 2.4 Data extraction

Data from the included studies were extracted into a standardized electronic spreadsheet developed for this review (see Supplementary Data C). The extracted data included: author, year, subject category (SWD, PE teachers, parents, other stakeholders), qualitative methods and theoretical perspectives, data collection and analysis, sample size, participant age, gender, disability type, and purpose of research and phenomena of interest.

### 2.5 Data analysis

The qualitative studies were analyzed using thematic synthesis, which is recognized as one of several methods for research synthesis, alongside meta-ethnography and meta-synthesis (44). The analyses were based on ontological relativism and epistemological constructionism, meaning that the researchers did not assume a singular, external, and knowable reality when interpreting the factors influencing SWD participation in PA within integrated schools. Instead, our values and experiences mediated and shaped our understanding of these factors (45).

This approach involved three stages: (1) thematic analysis, where integrated data were inductively coded into descriptive categories and themes, (2) thematic synthesis, where categories and themes were organized according to a conceptual framework, and (3) an interpretive synthesis of the thematic analysis and thematic synthesis results (27). XL and SH independently coded all text under “results” or “findings” headings, line by line, into descriptive categories and themes based on content and meaning. They then shared their codes and used a constant comparison method to compare and contrast them. The translation of concepts and ideas allowed for comparisons between studies while preserving the meaning of individual studies (46). A codebook was created, with codes refined and grouped into descriptive themes, followed by the development of analytical themes (44, 46).

As mentioned earlier, The BPN from SDT was selected as the theoretical framework for synthesizing themes (47). SDT posits that the three basic psychological needs are universal, and blocking any one of them can hinder autonomous engagement in behaviors—in this case, participation in PA. This framework served as the core factor model for understanding the dynamics within the PE environment, guiding the synthesis of themes and helping to recognize the interplay between environment, behavior, and motivation. Additionally, SDT helps recognize the interplay between environment, behavior, and motivation, and can be mapped onto the Behavior Change Wheel, aiding educational practitioners in identifying specific intervention strategies. Notably, while this theoretical framework replaced any *a priori* framework, the analytical method remained consistent with the proposed thematic synthesis approach.

Finally, XL and ZL completed the interpretive synthesis phase (44, 48) to explore relationships between themes identified during thematic analysis and synthesis. The goal of this interpretive synthesis was to provide a more conceptual understanding of attitudes, barriers, and facilitators to PA participation within the context of existing literature (44). The interpretive synthesis results were iteratively developed by the research team, allowing for the refinement, expansion, and confirmation of interpretive concepts, which encompassed the descriptive themes.

## 3 Result

### 3.1 Search results

**Figure 1:**
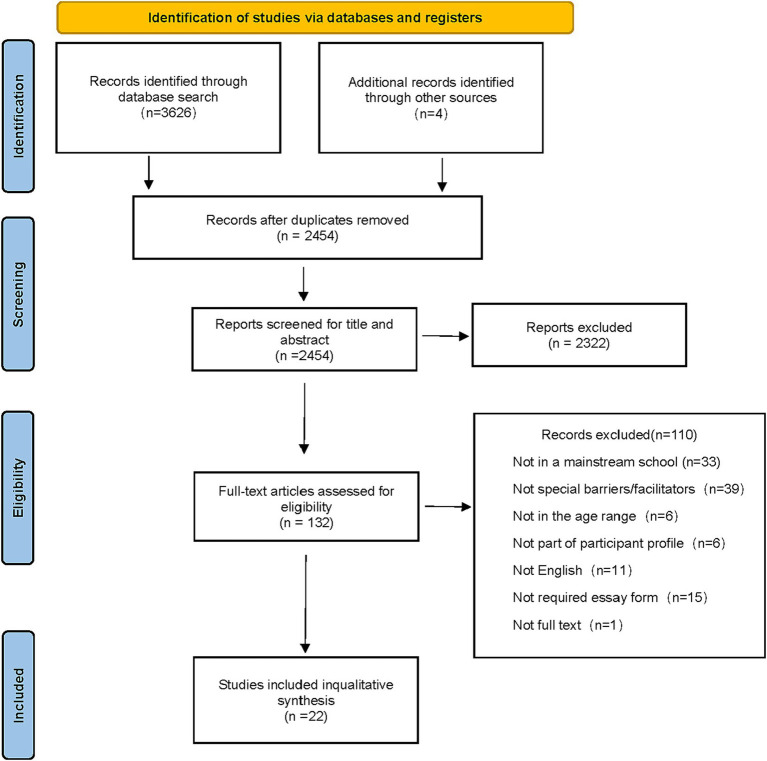
PRISMA flow diagram.

A total of 3,626 records were retrieved from the Web of Science, EBSCO (APA, CINAHI, ERIC), PubMed, and Scopus databases (see Supplementary Data A). Additionally, four articles were included from other sources that met the criteria. After removing 1,172 duplicates, 2,454 records were screened by title and abstract, with 132 meeting the criteria for full-text review. Ultimately, 22 studies were included, and 110 were excluded (see Figure 1).

In summary, 22 articles were included in this study. The main reasons for exclusion were: lack of relevant findings, quantitative article types, non-integrated school settings, participants not meeting requirements, unavailable literature, and non-English publications.

The qualitative data in all included studies were primarily collected through face-to-face interviews, semistructured audiotaped/telephone/video interviews, discussion groups, focus groups, field notes, reflective interview notes, and relational mapping. One study used an open-ended questionnaire (49). To ensure comprehensive data collection, multiple methods were often employed in many studies. Most studies analyzed data using thematic or content analysis, with specific qualitative methods like grounded theory, phenomenological, narrative approaches, and retrospective design. The results were presented as themes or categories, with five studies using theoretical models to aid analysis, such as the Social-Ecological Model (50), Self-Determination Theory (51), Lev Vygotsky’s Social Constructivism (52), Social Relational Model (53), and Occupational Socialization (49).

### 3.2 Characteristics of included studies

In terms of disability types, students with sensory impairments were the majority. Specifically, of the 22 studies, 7 focused on students with visual impairments (5, 19, 54–58), 7 focused on students with physical disabilities (21, 51, 59–63), and 2 involved students with cognitive disabilities (50, 64). The study subjects included individuals of various ages and identities, such as adults with physical disabilities (5, 19, 54, 58, 65), college students (21, 50, 59, 62), adolescents (53, 56, 57, 61, 63), and physically disabled athletes with multiple identities (51, 55, 60, 66). Additionally, the study included perspectives from other stakeholders, such as PE teachers (49, 52, 64, 67), adaptive teachers (67), parents (62, 66), rehabilitation clinicians (61), and support teachers (65). Aside from two studies (19, 55) that explored the intersections of gender, disability, and sports, and two studies that did not report gender (64, 67), all other studies included both male and female participants.

In the study contexts, experiences in mainstream schools primarily occurred in three settings: extracurricular leisure activities, fitness testing, and physical education classes. Most studies focused on the experiences of physical education in inclusive school settings (5, 19, 49, 52–57, 60, 62–65, 67). Three studies focused on SWD’s participation in sports during extracurricular time at school (21, 59, 66), three studies explored both curricular and extracurricular settings (50, 51, 61). Notably, Coates’ study also mentioned SWD’s experiences in school sports events (66), but due to limited details, these were not treated as a separate school context. Only one study specifically examined fitness testing (58).

It is noteworthy that four studies investigated the experiences of multiple stakeholders regarding SWD’s participation in PA (49, 62, 66, 67). For adults with disabilities, these studies typically used retrospective methods to recall past experiences in integrated educational settings. For athletes with physical disabilities, we are particularly interested in their experiences in integrated school settings. These details can be found in Supplementary Data C under “Phenomenon of Interest”.

### 3.3 Findings

**Figure 2:**
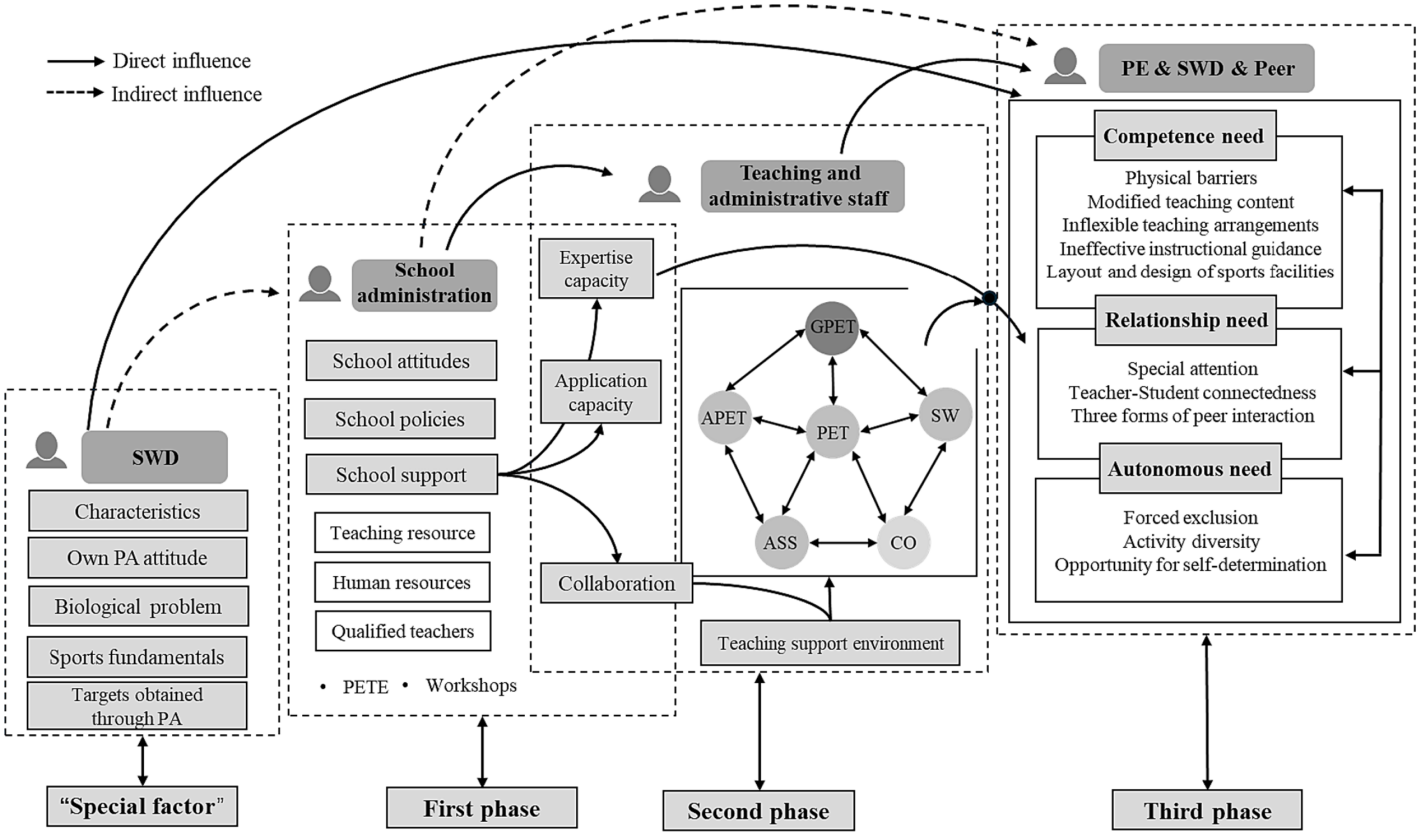
Theoretical map of factors influencing the participation of students with disabilities in physical activity in integrated school settings. SWD: Students with disabilities; PET: Physical Education Teacher (uniform designation for staff in the comprehensive school context in this study); GPET: General Physical Education Teacher; APET: Adapt ation of physical education teachers (specifically refers to teachers with adaptive knowledge and experience); ASS: Assistants in Physical Education (Include paraprofessional); SW: School workers; CO: Coaches at school recreation centres; PETE: physical education teacher education.

This study developed a dynamic theoretical model to explain factors affecting SWD participation in PA within mainstream schools, encompassing three scenarios: physical education classes, extracurricular leisure activities, and fitness testing (see Figure 2). The model categorizes influencing factors across three phases, from school administration, faculty/teaching and administrative staff, to SWD, covering 19 themes (see Supplementary Data D). This structure aims to foster “multi-directional” dialogue among different stakeholders within the school context. The *“First Phase”* involves school management, influencing the *“Second Phase,”* which consists of interactions between management and faculty. Themes like *“School Support”* from the *“First Phase”* impact faculty’s *“Expertise Capacity,” “Application Capacity,”* and *“Collaboration”* in the Second Phase. Effective faculty collaboration creates a *“Teaching Support Environment”* within the *“Second Phase.”* The *“Third Phase”* is the most direct and crucial for influencing SWD participation in PA, focusing on the interactions between faculty, SWD, and their peers. Factors in this phase are organized according to SDT’s basic psychological needs theory, divided into three sub-themes: autonomy needs, competence needs, and relatedness needs. These factors are dynamic and interrelated, allowing educational practitioners to tailor strategies to meet the specific needs of SWD.

The model also considers the students’ disability types, personal characteristics, and prior PA experience, which influence PA participation across all scenarios, particularly in the *“Third Phase”*.

#### 3.3.1 Special factor

SWD are unique individuals whose personal traits and other factors influence their behavior and likelihood of engaging in PA. Our findings highlight specific factors, including *“Biological problem,” “Physical capabilities,” “Personal characteristics,” “Attitudes toward PE,”* and *“Goals for participating in PA.”*

*“Biological problem,”* are often seen as major barriers to PA participation (68), especially concerning the severity of the disability. For example, one study noted that “*only our severely mentally handicapped are not in inclusion classes”* (49). The need for medical precautions due to existing conditions often requires reminders from various stakeholders to avoid exacerbating these conditions. As one participant with a heart condition mentioned, *“My doctor told me I cannot do high-intensity exercise, so I miss out on many opportunities for physical activity”* (53). Additionally, SWD with physical disabilities may require special equipment, which can also be a barrier to PA participation. For instance, a participant in a wheelchair expressed, *“It’s hard for me to jump, so I can only do warm-up activities”* (53).

However, some SWD with a strong foundation in PA can still participate in PE classes and even engage in high-intensity exercises despite their physiological limitations (64). These individuals often have high self-efficacy and do not let others’ opinions affect their participation. As one participant stated, *“Being a college student is about taking risks; I will not let others’ perceptions of what I can or cannot do stop me”* (59). Another affirmed, *“If I set my mind to it, I can do it”* (50). These participants also often display a strong desire to “prove themselves,” viewing PA as an opportunity to demonstrate that they are not different from others. This helps them feel like regular individuals and showcase their abilities (21, 59). Conversely, for more sensitive participants, engaging in PA can serve as a reminder of the differences between their current abilities and those they had before becoming disabled (21).

SWD’s attitudes towards PA also play a crucial role in their participation in integrated school settings (5, 21, 51, 55). These attitudes are largely shaped by past experiences with sports and their goals for engaging in PA. If SWD have had negative experiences, whether in school or elsewhere, these experiences can foster negative emotions. If such feelings are repeatedly reinforced, they may opt out of PA, perceiving it as meaningless. As one participant noted, *“I think that since I started to hate it, I did not try and get any meaning out of it. There was nothing I was open to learning”* (5).

The benefits of PA are well-documented, and some SWD continue to participate because it helps them stay healthy, manage their conditions, or maintain fitness (21, 50). Psychologically, engaging in PA can alleviate academic stress in a school environment (21, 50), and socially, it provides a platform for interaction and shared interests (21).

Notably, SWD’s personal interest in PA is significant, but it is not easily changed, especially since it is driven by intrinsic needs and experiences. Only a few participants indicated they do not engage in PA simply because they do not enjoy it: *“If I do not like it, I will not participate. I will not do something just because others do”* (5). *“I will not participate in activities I’m not interested in.”*

#### 3.3.2 “First phase” and “second phase”

The *“First phase”* and *“Second phase”* are not discussed as separate overarching themes. The *“First phase”* includes two major themes: *“School attitudes and policies”* and *“School support.” “School support”* encompasses sub-themes such as *“Teaching resources,” “Human resources,”* and *“Qualified teachers.”* These elements of *“School support”* directly influence the *“Second phase,”* affecting staff’s *“Expertise Capacity,” “Application Capacity,” and “Collaboration,”* Effective collaboration is crucial for creating a “*Supportive environment among staff*,” thereby becoming a facilitating factor.

##### 3.3.2.1 School attitudes and policies

The school’s attitudes shape policy directions and the administration’s commitment to implementing inclusive PE in integrated settings. Participants who are PE teachers believe school administrators should prioritize physical activity, highlighting its correlation with academic performance and setting clear evaluation criteria and goals. These objectives can guide teachers in supporting SWD. Currently, assessments are based solely on teachers’ discretion (67). They also acknowledge that the school’s inclusive policies provide a framework for developing teaching plans (52). For instance, SWD attending a recognized Special Olympics academy benefit from adapted PE courses offered weekly for 50 min throughout the semester (50).

Other participants noted that the school’s preference for competitive team sports and events can hinder SWD participation if a fair competitive environment and encouragement are not provided (66). For example, “*one school focuses heavily on rugby and basketball, where SWD often compete against much larger, able-bodied students, making it difficult for them to participate in fair competitions”*.

##### 3.3.2.2 School support

Most teacher participants expressed that insufficient school support hinders the implementation of inclusive teaching and SWD participation in PA. School support primarily involves providing teachers with adequate *“Teaching resources,” “Human resources,”* and *“Qualified teachers.”* It is important to note that while PE teachers are the primary facilitators of PA in schools, the staff involved in this phase includes assistant teachers, adaptive educators/APE, and coaches from school recreation centers.

Regarding support for PE resources, teachers feel they lack specialized adaptive equipment. *“These items become expensive once labeled for disabilities”* (67). Teachers believe that if schools provided this specialized equipment, it would greatly assist them in supporting students, helping to *“break down many barriers”* (67). Additionally, PE teachers report that classes including SWD often have low priority when scheduling gym facilities, with other classes occupying these spaces. Surprisingly, some PE teachers stated they have no input on student placement decisions, *“I am told when students will be included and that is about it”* (49). The school does not provide detailed information on SWD, such as specific injuries or conditions (52).

The large class sizes and diversity among students mean that class times are scheduled based on students’ timetables (49, 52). As a result, many teachers struggle with inadequate staffing, making it challenging to give students the attention they need. One teacher noted, *“Even though I prepared in advance, I am not confident in managing the increasing number and* var*iety of disabled students”* (52). Under these circumstances, PE teachers find it difficult to prepare outside of class, as they often have multiple responsibilities, including teaching other subjects. This workload makes it challenging to focus on the specific needs of SWD in their classes. *“Having a teaching assistant is helpful; when I assign activities to other students, the TA can supervise, allowing me to focus more on the disabled students”* (52). Even SWD rehabilitation specialists express dissatisfaction, believing that schools are not providing the inclusive PA opportunities they desire. One specialist remarked, *“Teachers are no longer PE specialists; they are generalists… certainly not offering specialized physical education”* (61).

Organizing training sessions and workshops on adaptive knowledge is a crucial way for schools to enhance the professional skills and expertise of PE teachers and other school staff. *“The workshops provided me with a basic understanding of how to teach students with disabilities in PE classes and made me aware of this issue, helping me to prepare better”* (52). However, teacher participants felt that the training workshops were inadequate*. “The lectures and consultations were not professional, and educational psychologists did not spend much time discussing the practical aspects of teaching students with disabilities”* (52). This limitation affects the practical application of the training in classroom settings.

Training for support staff and recreation center personnel is equally important. Some integrated schools provide support staff for PE classes, but their quality is not always ensured. For instance, one teacher noted, *“The assistants treat PE class as break time, sitting with unmotivated students. This becomes a problem, and even when reported to administration, no action is taken. It’s very disappointing”* (67). This suggests that for PE teachers, the effectiveness of inclusive PE teaching and student participation in PA is only possible if the school ensures support staff are properly trained and supervised. *“If we had appropriate training or a workshop for support staff at the start of the year, we could establish some basic rules”* (67). Similarly, SWD have expressed that during their free time at the school recreation center, staff lack adaptive knowledge due to insufficient training, preventing their participation in PA. *“The staff do not know simple ways to adapt activities, nor do they understand their importance to me,”* and *“I feel my need for accommodations is obvious, but staff question or are unwilling to provide them”* (59).

An often overlooked aspect of *“Support*” is training school staff on legal issues related to student injury and discrimination (66). Due to concerns about injury and potential lawsuits, as well as insufficient logistical support from the school, staff may be more inclined to exclude SWD from PA.

##### 3.3.2.3 Expertise and application capacity

The professional skills and practical application abilities of staff are primary influencing factors in the *“Third phase.”* Especially for general PE teachers, as the main implementers of education in integrated settings, their expertise significantly impacts students’ motivation to participate in PA both inside and outside the classroom (69). However, the reality is that most PE teachers lack sufficient training in adaptive knowledge during their undergraduate studies (70). This gap leaves them unprepared to effectively teach students with various disabilities (52). As some specialized APE teachers remarked, *“I do my best to include most of my students, but we do not always have the most qualified PE teachers”’* (49). For APE teachers, SWD feel they lack the knowledge and framework to make decisions and changes for their participation in PA, despite being professionals in disability. *“They are good at what they are supposed to do, mainly academic knowledge, but in PE classes, many of them lack any practical knowledge”* (57).

##### 3.3.2.4 Collaboration and teaching support environment

Effective collaboration helps create a supportive teaching environment among staff, which is a key factor in promoting the development of the*” Second “and “Third” phases.*

General PE teachers reported that they have actively sought advice from other school staff, such as social workers and APE teachers. Social workers are often more knowledgeable about the specific needs of special education students, making them a valuable resource. *“I often discuss potential solutions with the social worker”* (52). Some general PE teachers also consult with colleagues in other subjects who teach courses including SWD. *“I determine whether we can collaborate to fully meet the learning needs of students with disabilities”* (49). Similarly, due to their extensive and specialized knowledge, APE teachers are consulted by general PE teachers when planning basic motor skills tasks for SWD (49). Moreover, APE teachers regularly provide training for general PE teachers. Holding group meetings has been mentioned as an effective method for updating the knowledge base of PE teachers. *“At our school, we hold a group meeting at the start of each semester. We come together to discuss how to address the needs of students with disabilities, such as their types and characteristics, effective strategies, and next steps. After attending these meetings, I feel confident in teaching these students”* (52).

#### 3.3.3 Third phase

The *“Third phase”* is the central stage of this study’s findings, primarily derived from the “dialogue” between staff, SWD, and their peers. Researchers categorize the influencing factors of this phase into three major themes based on the SDT: *“Competence need,” “Relationship need,”* and *“Autonomous need”*.

##### 3.3.3.1 Competence need

The Competence need refers to an individual’s need to feel in control of their environment and experience mastery and effectiveness in activities (47). In integrated school settings, competence needs are reflected in four sub-themes: *“Physical barriers,” “Layout and design of sports facilities,” “Inflexible teaching arrangements and ineffective instruction,”* and *“Unmodified teaching content”*.

Before engaging in physical activities, SWD who require physical aids (such as wheelchairs) or have sensory impairments need the environment to be adapted to their physical limitations. For example, physical properties of assistive devices may conflict with unaltered classroom environments (53, 61, 64). SWD reported that their classrooms lacked elevators and had many stairs, making it difficult to reach PE classes without assistance. When classes are held on grassy fields, wheelchair users find it difficult to move around. During extracurricular activities, railings between gym doors hinder wheelchair access. Similarly, visually impaired participants expend more energy navigating to the school’s recreation center during their free time (21, 59) Due to large spaces and unsuitable building materials, they struggle to use auditory cues for orientation. *“The campus recreation center is not a welcoming or inclusive place because it is not designed for visually impaired individuals”* (59).

The layout and design of sports facilities are also crucial during PA. Particularly in PE environments, participants noted a characteristic feature: visibility. The sports environment is perceived as an unsafe space, especially when participants struggle with PA due to physical limitations in front of their peers, leading to feelings of insecurity and lack of confidence. In the PE teaching environment, the unsuitability of instructional tools, unmodified content, and teaching methods can exacerbate these anxieties, acting like a “magnifying glass.” Specifically, when instructional tools lack adaptive facilities that match SWD’s physical disabilities, they hinder task completion. For instance, for SWD with sensory impairments, the color (56) and size (53) of tools can provide basic, convenient preparation. The height and material of the tools can help SWD overcome psychological barriers, such as fear of jumping over high boxes (53). If SWD believe that general PE teachers cannot ensure their safety during instruction, they are more likely to fear participating in PA (19). Similar situations have been reported during extracurricular PA. At the school recreation center, facilities and layouts designed with sensory impairments in mind allow students to use auditory cues effectively. For those using physical aids, additional space between exercise equipment enables smooth movement, and smooth flooring facilitates wheelchair navigation (21, 59).

Inflexible teaching arrangements and ineffective instruction directly impact SWD’s perceived ability to participate in PA. Specifically, SWD noted that their teachers and coaches often lack flexibility in organizing and scheduling lessons (63), making it difficult for them to keep pace in PE classes (21). This issue is particularly pronounced for students with sensory impairments, who find it challenging to understand and follow verbal instructions, especially with limited practice time (53). The failure to acquire skills can lead to frustration. It is evident that SWD need assistance during class. Providing additional support and guidance, such as timely correction of movements (53), the involvement of support staff (49, 52, 53), or assigning a peer buddy (53) can significantly improve the situation. For students with sensory impairments, tactile modeling and physical guidance are considered crucial instructional supports. *“I told the teacher she could touch me; tactile demonstrations and physical guidance are helpful for me”* (54). These supports are also applicable during fitness tests. Without proper guidance on the feasibility of certain exercises, students may not perform the correct movements, limiting their potential (58).

Adapting the curriculum is crucial for helping SWD develop their abilities. If the curriculum is not reasonably modified to meet the specific needs of SWD, it can lead to uncomfortable situations, such as *“being dragged along by a caregiver”* (64). Unmodified content can make SWD feel that the material is too complex (53), resulting in negative experiences of not knowing how to proceed or complete tasks properly, as expressed by students: *“I do not know what to do” and “I do not understand the format”* (51, 56). SWD with less apparent disabilities, especially those who do not use wheelchairs, have reported that teachers often overlook their physical limitations. They are required to perform the same activities as their peers, which can result in physical pain (63).

Notably, one SWD emphasized that only modifications that truly meet their needs are considered necessary. They criticized superficial adjustments, likening them to “band-aid” fixes. They felt that changes made in PE classes often do not consider their specific needs, only minimally enabling participation (57). For example, SWD attending Special Olympics schools find Paralympic sports curriculum meaningful. However, in reality, these courses are often tokenistic, with content offered once a year, reinforcing the notion that SWD are less capable in PA compared to non-SWD (64).

##### 3.3.3.2 Relationship need

The Relationship need refers to an individual’s sense of connection and support from others in their environment (47). In integrated school settings, this need is reflected in three areas: *“Teacher-student connectedness,” “Three types of peer interactions,”* and *“Special attention”*.

Teacher-student interactions and communication play a crucial role in integrating SWD into mainstream PE classes. These interactions help SWD feel treated equally (5) and cared for Haegele et al. (57), fostering positive emotions such as verbal encouragement (19, 53). The genuine feelings of students in class are critical. Some SWD feel they are only “obligated participants,” believing that teachers are not genuinely concerned with their learning or performance, merely going through the motions to award credits (55). One student remarked, *“My presence in class is just a legal requirement”* (57). This lack of care can lead to negative outcomes, such as reduced expectations for participating in PA (19). Participants also acknowledged that communication serves as a “bridge” in teacher-student interactions. When communication is one-sided, students can feel frustrated, as one noted, *“Constantly asking for changes is exhausting, and my patience is limited”* (57). Two-way communication allows teachers to seek SWD’s opinions and for SWD to share their circumstances and suggestions. This reciprocal relationship fosters mutual dependence, enhancing the decision-making and problem-solving process (54, 56, 57, 60).

Inclusion in integrated school environments increases interactions between SWD and their peers, which can be categorized into three types: friendships, positive interactions, and negative interactions (71). The first two types are beneficial for social skill development. Positive interactions are frequently mentioned in integrated school settings. SWD often say that participating in school PA gives them topics to discuss with others (21) and even to bond over complaints, which helps bring peers closer. They may engage in PA to socialize with friends (21, 59). For some SWD, the company of friends is a primary motivator for participation: *“If I cannot do it with friends, I’m not interested.”* Positive peer interactions not only assist participants in completing activities—*"When cycling, my peer describes the surroundings to me”* (21)—but also foster a sense of belonging, especially when SWD form friendships with peers. For instance, SWD who joined a campus club they enjoyed felt positive interactions and respect within a group of like-minded individuals, saying, *“I was treated as a group member,” and “I could give my opinion on where we should go”* (59).

Unfortunately, many SWD also report negative experiences in PA, including isolation (66) and bullying, such as verbal teasing (56), mocking (52), being given derogatory nicknames—*"My visual impairment makes me an easy target”* (19)—and even physical violence (56). During team games, SWD’s “limitations” are often magnified. Many participants reported being excluded by peers, who would ignore them (5, 56, 61). Without teacher intervention, peers often showed reluctance, even through body language, to play with SWD (52). SWD’s abilities were sometimes seen as a burden to winning (52, 53). One student said, *“I’m always picked last, it’s always between the overweight guy and me”* (5). Others felt that having SWD on their team made games less enjoyable (53).

An often overlooked external barrier affecting SWD-peer interactions is the interruption caused by private paraeducator. During group activities in PE, excessive intervention by aides disrupts interactions between SWD and other students. As one observation noted, *“Students find it annoying when adults older than them are involved”* (65).

*“Special attention”* during uncomfortable social interactions is a barrier to SWD’s participation in PA, as it highlights their differences from others. SWD often feel watched during gym activities or fitness tests, describing themselves as *“standing out like a sore thumb”* (21, 58). Additionally, when teachers treat SWD in an overly special manner (5, 58, 60), it inadvertently draws more attention from peers. For instance, some SWD recall receiving special rewards from teachers for completing basic tasks (5). In recreation centers, coaches may announce the arrival of a “special” participant to the entire class (59). During fitness tests, requiring the class to wait for SWD to finish before being dismissed makes SWD feel like “museum exhibits” (58). Such differential treatment underscores their differences. This behavior, often referred to as the “hero syndrome” (21), occurs when school staff or strangers exaggerate their praise for SWD during routine physical activities (59). Such reactions can convey that the SWD have accomplished something extraordinary, reinforcing the notion that their participation is exceptional.

##### 3.3.3.3 Autonomous need

*“Autonomous need”* refers to the degree of self-determination an individual experiences in their actions (47). This concept is reflected in three subthemes: *“Forced exclusion,” “Activity diversity,”* and *“Opportunities for self-determination”*.

*“Forced exclusion”* and *“Opportunities for self-determination”* are frequently discussed. For most SWD, although PE is often mandatory, they lack the autonomy to choose whether or how to participate in PA. While the official rationale is often safety concerns, the underlying issue is the low expectations staff have for SWD’s ability to complete PA. In PE classes, they are often forced into alternative activities (5, 19, 60, 63, 67), such as simple ball tossing in a separate space (5, 19), running on a treadmill (56), exercising only with their aides (63), or being assigned non-physical tasks like writing assignments or acting as referees (53, 62, 67). SWD have reported similar experiences during extracurricular activities at school recreation centers and fitness tests, expressing that they often do not participate but instead sit and watch (58, 59, 66).

Similarly, the low expectations of SWD’s personal aides can lead to prohibiting their participation in PE without consulting them (63, 65). For SWD who require assistance to participate in PA, unhelpful or inactive aides can indirectly prevent their involvement (65).

However, outside PE classes, where teachers exert significant control, SWD generally have more autonomy. Although university SWD often report that heavy coursework and limited free time restrict their ability to decide when to exercise (50), university recreation centers offer systems for reserving sports facilities, helping alleviate this issue. This allows SWD to schedule their activities according to their availability (50). Recreation centers also offer a variety of facilities and classes, promoting greater choice in PA participation. However, some SWD have reported that, despite paying the same fees, they find few activities suited to their abilities, which limits their willingness to engage in PA (21).

## 4 Discussions

An interesting observation in this study, which aimed to include factors affecting SWD participation in PA within integrated school environments, is that most scenarios described in the 22 included articles occurred in PE classes. Few studies addressed extracurricular and fitness test contexts, and sports day experiences were only briefly mentioned (66). This may be due to the nature of these contexts: PE is a mandatory course in integrated settings for SWD, and fitness tests are often considered compulsory, forming part of the PE curriculum (72). Although fitness testing is a common practice in PE worldwide, few studies have explored students’ perceptions of participating in these tests (73). The extracurricular activities analyzed in this study primarily involved university and athlete SWD. Outside PE classes, other SWD, often spend their time in family and social settings. Athlete SWD may also participate in sports teams or clubs outside school. For university SWD, the campus environment resembles a “small society,” where they have relative autonomy. The limited research on extracurricular leisure activities may be explained by the social construction theory, which suggests society often assigns negative meanings to disability (74). Consequently, researchers and staff planning and studying campus extracurricular services may assume SWD are less active participants outside mandatory settings like PE. However, the use of campus recreation centers by SWD for extracurricular PA is an important area of research. Previous studies have compared the psychosocial variables of students who use recreational facilities with those who do not. Users tend to feel more at home on campus, have better social skills, and experience a higher quality of life (75). This does not imply that all schools, such as middle schools, can provide facilities similar to university recreation centers. However, applying universal design principles to eliminate physical and environmental barriers (76) and exploring ways to provide more sports facilities and activities can promote SWD’s participation in extracurricular PA in integrated settings. This warrants further research.

This study synthesized qualitative research from diverse stakeholders to identify factors influencing SWD’s participation in PA within integrated school settings. The results section presents a dynamic theoretical model with a *“special factor”* and three stages, developed through inductive and deductive reasoning. The model highlights that no single factor can fully explain the extent of SWD’s participation in PA. Educators must consider both the physiological, behavioral, and cognitive factors of SWD (see *“special factor”*) and the mediating variables and processes within the school environment (the three stages) that shape their behavior. The social model of relationships supports this, suggesting that both individual impairments and environmental factors contribute to barriers (77). Specifically, influencing SWD participation in integrated school settings involves a top-down process. This includes structural changes in schools, such as enhanced teacher training, resource allocation, and curriculum design, which influence staff behaviors that either facilitate or hinder SWD participation in PE and extracurricular activities. Understanding these dynamics is essential for effectively promoting SWD’s active participation in PA.

The perspectives presented in this study are significant. While we agree with Barton’s view that *“the responsibility for change should not be placed on disabled students but on the education system to reform”* (78), this study also recognizes the role of individual factors in SWD participation. Previous research has shown that factors such as confidence, self-efficacy, health status (e.g., injuries, disabilities, medical conditions, obesity), and economic issues impact the exercise behaviors for both disabled and non-disabled university students (79, 80). This implies that the individual factors identified in the *“Special factor”* not only occur in integrated school settings but also influence behaviors in all other contexts as a “common factor.” Although individual factors are not the core focus of this study, their impact is acknowledged. We suggest that future research could delve deeper into these internal personal factors.

In the first two stages of the model, we explore how school administrators influence SWD participation in PA from a top-down perspective. Although their influence is not directly affect by SWD, it significantly impacts faculty members. Participants highlighted the “illusion” of inclusivity in integrated schools, where administrators often mistakenly equate “integration” with “inclusion.” However, inclusion goes beyond providing access to integrated schools for previously excluded students. It is an ongoing process aimed at enhancing learning participation for all students to meet their educational needs (81). Contrary to the findings of this study, participants in the *“Third stage”* observed that SWD in integrated school settings often participate out of obligation. In PE classes, they merely attend alongside peers without meaningful adjustments in content or curriculum, and any modifications made by PE teachers are superficial. This contradicts the philosophy of inclusive education, which advocates for diverse learning methods and reducing the burden on students to adapt their learning styles (82). Similarly, in extracurricular PA, inclusive education requires school staff to provide services that ensure all students, regardless of ability, can reach their full potential (83). Such “pseudo-inclusion” prevents staff from receiving adequate support and hinders SWD from achieving meaningful learning experiences or social interaction with non-disabled peers (5, 18, 19).

Fully integrating SWD into any subject area can be challenging. Additionally, complete integration into activity environments presents unique challenges for school staff, particularly PE teachers (84). This study highlights various roles that influence SWD participation in PA, including PE teachers, APE specialists, recreation center coaches, and teaching assistants, without distinguishing their specific differences. However, PE teachers are the primary implementers in PE settings. In the model, PE teachers are positioned in a middle layer. They are constrained by the support and environment provided by school administrators and face the challenged of accommodating various disability types in PA. Their professional competence and practical knowledge application are crucial components in delivering inclusive education to SWD.

In reality, most PE teachers do not receive systematic training in adaptive knowledge or practical experience during their undergraduate studies. University PE programs rarely include disability content, and when they do, it is often from a student perspective rather than a professional teaching standpoint. Pre-service training for including SWD in regular PE is limited (70). PE teacher education programs and professional development workshops are primary avenues for enhancing their self-efficacy in educating SWD. These resources, as highlighted in the study, play a critical role in equipping PE staff with the knowledge and strategies necessary for inclusive practices (85). Although participants noted that APE lectures provided by their schools lacked professionalism and practicality, the adaptive knowledge discussed did help prepare them for teaching. This underscores the importance of professional workshops and physical education teacher education (PETE) programs in guiding effective PE implementation. This finding aligns with previous research, where Beamer and Yun found a significant correlation between PE staff’s inclusive training and their inclusive behaviors (86). Improving pre-service teachers’ instructional behaviors is crucial for promoting inclusivity (85). Without sufficient adaptive knowledge, teachers may struggle to implement inclusive education, which can negatively impact their attitudes (87), and reduce SWD participation in PA.

Training should not be limited to front-line teachers but must also include support staff, a point emphasized in the study’s findings but often overlooked in current research. Teachers frequently see support staff as assisting both students and teachers by ensuring safety and managing behavior (88). However, the research indicates that support staff lack sufficient training and subject-specific knowledge, making it harder for them to effectively support PE teachers and create an inclusive environment for SWD. This aligns with findings from Haycock and Smith, who reported that the limited skills, knowledge, and expertise of support staff can hinder their ability to meet students’ needs (89). Vickerman and Blundell reported that among 142 surveyed support staff, 63.3% received general training, while only 5.5% received specialized PE training (90). Optimal practices occur only when teachers and support staff collaborate in planning and implementing PE lessons.

The third stage of the model applied the SDT framework, focusing on three basic psychological needs (91) to analyze factors affecting SWD participation in PA and their dynamic relationships. Specifically, when these three needs are met, individuals are more likely to engage in behaviors voluntarily. This framework provides clear guidance for creating a supportive environment. Previous studies have also used the social-ecological model to identify key themes in this area (50, 92).

Successfully implementing inclusive education involves meeting the diverse learning styles and educational needs of students while providing necessary support (6, 8). The third stage reveals that integrated schools and educators often fail to provide a supportive environment for the educational needs of SWD. Among the three basic needs, factors affecting the need for competence are most prevalent. This aligns with scholars’ critiques of the dominance of physical ability in integrated settings (93, 94), where PE curricula and practices overly emphasize normative standards of ability, performance, and competitiveness (95). In PE classes, extracurricular activities, or fitness testing, most SWD reported experiencing an environment of dominated by “ableism”. Traditional assessments and standardized tests recognize ability, implying that SWD’s abilities can be measured, categorized, and judged. Ability is perceived as a fixed attribute that students either have or lack (96). Evidently, SWD are often classified as lacking ability within environments dominated by traditional content (97) Some SWD attempt to demonstrate their adapted movements to teachers or coaches to showcase their abilities. However, educators often value knowledge related only to non-disabled bodies and traditional physical expressions, neglecting the physical cultures and abilities of disabled individuals.

“Ableism,” a form of subtle discrimination, is rooted in social constructs. Challenging this construct is complex, as it requires mechanisms supported by national ideologies and political institutions (98). Educators, as frontline practitioners, should actively challenge stereotypes and societal barriers. Actions like organizing Paralympic school days (99), incorporating disability sports units into PE classes (100), and promoting open communication can help. Such actions can reduce stigma and exclusion of SWD (101). However, as Maher noted, these attempts at empathy can be complex and sometimes inadequate. They are typically short-term and fully understand the experience of disability without living it firsthand remains challenging (102). Future research should address this issue and explore practical solutions.

## 5 Limitations

The majority of included studies focused on physical education contexts, with limited exploration of leisure PA and fitness testing environments. This uneven focus may have led to an overrepresentation of barriers and facilitators relevant to structured school settings, while underrepresenting informal or recreational contexts.

This study used broad terminology when referring to “students with disabilities” and did not focus on any specific type of disability. As a result, the findings may not be generalizable to individuals with every specific type of disability.

The inclusion of only English-language articles may have excluded relevant studies published in other languages, potentially narrowing the diversity of perspectives and limiting the cultural applicability of the findings.

The inclusion of qualitative research in this study means that its findings are influenced by the nature of qualitative methods. The scope and depth of qualitative results are often limited by factors such as the number of participants, their sociodemographic characteristics, the local context of data collection, and the lack of integration of inclusive sports experiences and global perspectives. Additionally, the chosen qualitative paradigms and theoretical frameworks shaped the scope and nature of the investigation as well as the researchers’ interpretations (103).

Studies conducted over extended timeframes or in varying sociocultural contexts may reflect evolving practices, policies, or attitudes, potentially impacting the comparability and consistency of findings.

## 6 Conclusion

This study developed a dynamic theoretical model comprising a *“Special factor”* and three stages, suggesting that no single factor can fully explain the extent of SWD participation in PA within integrated school environments. Educational practitioners must understand not only the physiological, behavioral, and cognitive factors of SWD (see *“Special factor”*) but also the intermediary factors and processes in integrated school settings that influence their behavior (three stages). The policies and support provided by school management influence the professional and practical capabilities of staff and the development of a supportive environment. These three factors directly impact the interactions between staff and SWD. The final stage uses the SDT’s basic psychological needs as a theoretical foundation, categorizing the factors influencing SWD participation in PA in three integrated school contexts (physical education, fitness testing, and extracurricular sports) into three themes/needs. It reveals that SWD participation in PA requires comprehensive support from personnel in a dynamic collaboration process. Support staff can develop strategies to address specific factors and create an environment that supports these needs.

## 7 Implications for future research

Future research, particularly longitudinal or intervention-based studies, could explicitly test the proposed strategies in this study to assess their practical impact on promoting the participation of SWD in physical activities. These studies would provide crucial evidence on the effectiveness of the strategies within the dynamic model, helping to refine and validate the proposed interventions. The model highlights the need for comprehensive support that includes not only school administrators’ policies but also teachers’ collaboration and the overall school environment, which together influence SWD participation in physical education, fitness testing, and extracurricular activities.

The third stage of the model, based on SDT, emphasizes the importance of fulfilling the basic psychological needs of competence, relatedness, and autonomy to foster engagement. Future research should explore how these needs can be effectively addressed in different school contexts, testing specific interventions to create an inclusive environment that enhances SWD’s participation in physical activities. By investigating these strategies through longitudinal or intervention-based studies, researchers can provide more concrete recommendations for improving SWD participation in integrated school settings.

## Data Availability

The original contributions presented in the study are included in the article/Supplementary material, further inquiries can be directed to the corresponding author.
