# Supplementary material for: Barriers and facilitators to participation in physical activity for students with disabilities in an integrated school setting: a meta-synthesis of qualitative research evidence
Source: Front Public Health. 2025 Mar 12;13:1496631. doi: 10.3389/fpubh.2025.1496631 (PMC11936882; doi:10.3389/fpubh.2025.1496631)
Supplement: Supplementary file 1 [file Table_1.docx]

Supplementary Material

Supplementary Data A: Search Strategies.

**PubMed**

| **Searches** | **Search terms (AB/TI, MeSH -2024)** | **Results** |
| --- | --- | --- |
| **#1** | ("college*"[MeSH Terms] OR "school*"[MeSH Terms] OR "physical education*"[MeSH Terms] OR "college*"[Title/Abstract] OR "university*"[Title/Abstract] OR "academy*"[Title/Abstract] OR "school*"[Title/Abstract] OR "high school*"[Title/Abstract] OR "physical education*"[Title/Abstract] OR "inclusive education"[Title/Abstract] OR "middle school*"[Title/Abstract]） | **964,029**  **985035**  **38,437** |
| **#2** | （"intellectual disabilit*"[MeSH Terms] OR "disabled person*"[MeSH Terms] OR "disabled children*"[MeSH Terms] OR "Sports for Persons with Disabilities"[MeSH Terms] OR "physical disabilit*"[Title/Abstract] OR "vision disabilit*"[Title/Abstract] OR "childhood onset disabilit*"[Title/Abstract] OR "motor disabilit*"[Title/Abstract] OR "vision impairment"[Title/Abstract] OR "intellectual disabilit*"[Title/Abstract] OR "hearing impairment"[Title/Abstract] OR "hearing disabilit*"[Title/Abstract] OR "multiple disabiliti*"[Title/Abstract] OR "disabilit*"[Title/Abstract] OR "impairment*"[Title/Abstract] OR "disabled person*"[Title/Abstract] OR "disabled children*"[Title/Abstract] OR "disabled student*"[Title/Abstract] OR "extremity disability"[Title/Abstract] OR "upper extremity disability"[Title/Abstract] OR "lower extremity disability"[Title/Abstract] OR "wheelchair"[Title/Abstract] OR "Sports for Persons with Disabilities"[Title/Abstract]） | **778,775**  **810,225**  **33550** |
| **#3** | （"facilitat*"[MeSH Terms] OR "attitude*"[MeSH Terms] OR "motivat*"[MeSH Terms] OR"influen*"[MeSH Terms] OR "hobbies*" [MeSH Terms] OR "barrier*"[Title/Abstract] OR "facilitat*"[Title/Abstract] OR "attitude*"[Title/Abstract] OR "Motivat*"[Title/Abstract] OR "influen*"[Title/Abstract] OR "belief*"[Title/Abstract] OR "interest*"[Title/Abstract] OR "hobb*"[Title/Abstract] OR"factor"[Title/Abstract]） | **6,375,044**  **6,574,457**  **261,465** |
| **#4** | （"exercise*"[MeSH Terms] OR "sport*"[MeSH Terms] OR "physical activit*"[Title/Abstract] OR "exercise*"[Title/Abstract] OR "sport*"[Title/Abstract]） | **747,476**  **775826**  **28,350** |
| **#5** | （"student*"[MeSH Terms] OR "school teacher*"[MeSH Terms] OR "adult*"[MeSH Terms] OR "adolescent*"[MeSH Terms] OR "adult children*"[MeSH Terms] OR "student*"[Title/Abstract] OR "junior*"[Title/Abstract] OR "undergraduate*"[Title/Abstract] OR "university*"[Title/Abstract] OR "teacher*"[Title/Abstract] OR "adult*"[Title/Abstract] OR "adolescent*"[Title/Abstract] OR "teenager*"[Title/Abstract] OR "young adult*"[Title/Abstract] OR "adult children*"[Title/Abstract] OR "teen*"[Title/Abstract] OR "young people"[Title/Abstract] OR "youth"[Title/Abstract] OR "girls"[Title/Abstract] OR "boys"[Title/Abstract] OR "young women"[Title/Abstract] OR "young men"[Title/Abstract]） | **9975140**  **10158622**  **183,482** |
| **#6** | #1 AND #2 AND #3 AND #4 AND #5 | **887** |

**Web of Science**

| **Searches** | **Search terms (AB, TI, TS -2024)** | **Results** |
| --- | --- | --- |
| **#1** | ((TS=("college*" OR "university*" OR "academy*" OR "school*" OR "high school*" OR "physical education*" OR "inclusive education" OR "middle school*")) OR TI=("college*" OR "university*" OR "academy*" OR "school*" OR "high school*" OR "physical education*" OR "inclusive education" OR "middle school*")) OR AB=("college*" OR "university*" OR "academy*" OR "school*" OR "high school*" OR "physical education*" OR "inclusive education" OR "middle school*") | **3519265**  **4139841**  **614070** |
| **#2** | ((TS=("physical disabilit*" OR "vision disabilit*" OR "childhood onset disabilit*" OR "motor disabilit*" OR "vision impairment" OR "intellectual disabilit*" OR "hearing impairment" OR "hearing disabilit*" OR "multiple disabiliti*" OR "disabilit*" OR "impairment*" OR "disabled person" OR "disabled children" OR "disabled student" OR "extremity disability" OR "upper extremity disability" OR "lower extremity disability" OR "wheelchair" OR "Sports for Persons with Disabilities")) OR TI=("physical disabilit*" OR "vision disabilit*" OR "childhood onset disabilit*" OR "motor disabilit*" OR "vision impairment" OR "intellectual disabilit*" OR "hearing impairment" OR "hearing disabilit*" OR "multiple disabiliti*" OR "disabilit*" OR "impairment*" OR "disabled person" OR "disabled children" OR "disabled student" OR "extremity disability" OR "upper extremity disability" OR "lower extremity disability" OR "wheelchair" OR "Sports for Persons with Disabilities")) OR AB=("physical disabilit*" OR "vision disabilit*" OR "childhood onset disabilit*" OR "motor disabilit*" OR "vision impairment" OR "intellectual disabilit*" OR "hearing impairment" OR "hearing disabilit*" OR "multiple disabiliti*" OR "disabilit*" OR "impairment*" OR "disabled person" OR "disabled children" OR "disabled student" OR "extremity disability" OR "upper extremity disability" OR "lower extremity disability" OR "wheelchair" OR "Sports for Persons with Disabilities") | **1327780**  **1586845**  **259065** |
| **#3** | ((TS=("barrier*" OR "facilitat*" OR "attitude*" OR "Motivat*" OR "influen*" OR "belief*" OR "interest*" OR "hobby*" OR"factor")) OR TI=("barrier*" OR "facilitat*" OR "attitude*" OR "Motivat*" OR "influen*" OR "belief*" OR "interest*" OR "hobby*" OR"factor")) OR AB=("barrier*" OR "facilitat*" OR "attitude*" OR "Motivat*" OR "influen*" OR "belief*" OR "interest*" OR "hobby*" OR"factor") | **21410024**  **23620697**  **2210673** |
| **#4** | ((TS=("physical activit*" OR "exercise*" OR "sport*")) OR TI=("physical activit*" OR "exercise*" OR "sport*")) OR AB=("physical activit*" OR "exercise*" OR "sport*") | **1824045**  **2003223**  **179178** |
| **#5** | ((TS=("student*" OR junior* OR "undergraduate*" OR "university*" OR "teacher*" OR "adult*" OR "adolescent*" OR "teenager*" OR "young adult*" OR "physical teacher*" OR "adult children*" OR teen* "OR" "young people" OR "youth" OR "girls" OR "boys" OR "young women" OR "young men")) OR TI=("student*" OR junior* OR "undergraduate*" OR "university*" OR "teacher*" OR "adult*" OR "adolescent*" OR "teenager*" OR "young adult*" OR "physical teacher*" OR "adult children*" OR teen* "OR" "young people" OR "youth" OR "girls" OR "boys" OR "young women" OR "young men")) OR AB=("student*" OR junior* OR "undergraduate*" OR "university*" OR "teacher*" OR "adult*" OR "adolescent*" OR "teenager*" OR "young adult*" OR "physical teacher*" OR "adult children*" OR teen* "OR" "young people" OR "youth" OR "girls" OR "boys" OR "young women" OR "young men") | **12644918**  **13875472**  **1230554** |
| **#6** | #1 AND #2 AND #3 AND #4 AND#5 | **3489** |

**SCOPUS**

| **Searches** | **Search terms (AB/TI/KEYWORD -2024)** | **Results** |
| --- | --- | --- |
| **#1** | ("college*" OR "university*" OR "academy*" OR "school*" OR "high school*" OR "physical education*" OR "inclusive education" OR "middle school*") | **3798085**  **147138**  **3,945,223** |
| **#2** | ("physical disabilit*" OR "vision disabilit*" OR "childhood onset disabilit*" OR "motor disabilit*" OR "vision impairment" OR "intellectual disabilit*" OR "hearing impairment" OR "hearing disabilit*" OR "multiple disabiliti*" OR "disabilit*" OR "impairment*" OR "disabled person" OR "disabled children" OR "disabled student" OR "extremity disability" OR "upper extremity disability" OR "lower extremity disability" OR "wheelchair" OR "Sports for Persons with Disabilities") | **1,152,858**  **55538**  **1,208,396** |
| **#3** | ("barrier*" OR "facilitat*" OR "attitude*" OR "Motivat*" OR "influen*" OR "belief*" OR "interest*" OR "hobby*" OR"factor") | **20,847,358**  **969526**  **21,816,884** |
| **#4** | ("physical activit*" OR "exercise*" OR "sport*") | **1,259,631**  **62595**  **1,322,226** |
| **#5** | ("student*" OR junior* OR "undergraduate*" OR "university*" OR "teacher*" OR "adult*" OR "adolescent*" OR "teenager*" OR "young adult*" OR "physical teacher*" OR "adult children*" OR teen* "OR" "young people" OR "youth" OR "girls" OR "boys" OR "young women" OR "young men") | **165,959**  **7219**  **173,178** |
| **#6** | #1 AND #2 AND #3 AND #4 AND #5 | **208** |

**ERIC**

| **Searches** | **Search terms (AB, TI, DE -2024)** | **Results** |
| --- | --- | --- |
| **#1** | (DE "school*") OR TI ( "college*" OR "university*" OR "academy*" OR "school*" OR "high school*" OR "physical education*" OR "inclusive education" OR "middle school*" ) OR AB ("college*" OR "university*" OR "academy*" OR "school*" OR "high school*" OR "physical education*" OR "inclusive education" OR "middle school*") | **882,120**  **9759**  **891879** |
| **#2** | (DE "disabilities or disabled") OR TI ("physical disabilit*" OR "vision disabilit*" OR "childhood onset disabilit*" OR "motor disabilit*" OR "vision impairment" OR "intellectual disabilit*" OR "hearing impairment" OR "hearing disabilit*" OR "multiple disabiliti*" OR "disabilit*" OR "impairment*" OR "disabled person" OR "disabled children" OR "disabled student" OR "extremity disability" OR "upper extremity disability" OR "lower extremity disability" OR "wheelchair" OR "Sports for Persons with "Disabilities") OR AB ("physical disabilit*" OR "vision disabilit*" OR "childhood onset disabilit*" OR "motor disabilit*" OR "vision impairment" OR "intellectual disabilit*" OR "hearing impairment" OR "hearing disabilit*" OR "multiple disabiliti*" OR "disabilit*" OR "impairment*" OR "disabled person" OR "disabled children" OR "disabled student" OR "extremity disability" OR "upper extremity disability" OR "lower extremity disability" OR "wheelchair" OR "Sports for Persons with "Disabilities") | **29952**  **731**  **30683** |
| **#3** | (DE "attitude*") OR (DE "Motivat*")) OR (DE "influen*") OR (DE "belief*") OR (DE "interest*") OR TI ("barrier*" OR "facilitat*" OR "attitude*" OR "Motivat*" OR "influen*" OR "belief*" OR "interest*" OR "hobby*"OR "factor") OR AB ("barrier*" OR "facilitat*" OR "attitude*" OR "Motivat*" OR "influen*" OR "belief*" OR "interest*" OR "hobby*"OR "factor") | **505,417**  **9193**  **514610** |
| **#4** | (DE "exercise*") OR TI （"physical activit*" OR "exercise*" OR "sport*"） OR AB （"physical activit*" OR "exercise*" OR "sport*"） | **56458**  **647**  **57105** |
| **#5** | (DE "student*") OR (DE "teacher*")) OR (DE "young adult*") OR (DE "youth") OR TI ("student*" OR junior* OR "undergraduate*" OR "university*" OR "teacher*" OR "adult*" OR "adolescent*" OR "teenager*" OR "young adult*" OR "physical teacher*" OR "adult children*" OR teen* "OR" "young people" OR "youth" OR "girls" OR "boys" OR "young women" OR "young men") OR AB ("student*" OR junior* OR "undergraduate*" OR "university*" OR "teacher*" OR "adult*" OR "adolescent*" OR "teenager*" OR "young adult*" OR "physical teacher*" OR "adult children*" OR teen* "OR" "young people" OR "youth" OR "girls" OR "boys" OR "young women" OR "young men") | **1239512**  **14566**  **1254078** |
| **#6** | #1 AND #2 AND #3 AND #4 AND #5 | **135** |

** Searches made through EBSCO-host*

**CINAHL Complete**

| **Searches** | **Search terms (AB, TI, DE 2024)** | **Results** |
| --- | --- | --- |
| **#1** | (DE "school*") OR TI ( "college*" OR "university*" OR "academy*" OR "school*" OR "high school*" OR "physical education*" OR "inclusive education" OR "middle school*" ) OR AB ("college*" OR "university*" OR "academy*" OR "school*" OR "high school*" OR "physical education*" OR "inclusive education" OR "middle school*") | **435,360**  **8760**  **444120** |
| **#2** | (DE "disabilities or disabled") OR TI ("physical disabilit*" OR "vision disabilit*" OR "childhood onset disabilit*" OR "motor disabilit*" OR "vision impairment" OR "intellectual disabilit*" OR "hearing impairment" OR "hearing disabilit*" OR "multiple disabiliti*" OR "disabilit*" OR "impairment*" OR "disabled person" OR "disabled children" OR "disabled student" OR "extremity disability" OR "upper extremity disability" OR "lower extremity disability" OR "wheelchair" OR "Sports for Persons with "Disabilities") OR AB ("physical disabilit*" OR "vision disabilit*" OR "childhood onset disabilit*" OR "motor disabilit*" OR "vision impairment" OR "intellectual disabilit*" OR "hearing impairment" OR "hearing disabilit*" OR "multiple disabiliti*" OR "disabilit*" OR "impairment*" OR "disabled person" OR "disabled children" OR "disabled student" OR "extremity disability" OR "upper extremity disability" OR "lower extremity disability" OR "wheelchair" OR "Sports for Persons with "Disabilities") | **81389**  **2644**  **84033** |
| **#3** | (DE "attitude*") OR (DE "Motivat*")) OR (DE "influen*") OR (DE "belief*") OR (DE "interest*") OR TI ("barrier*" OR "facilitat*" OR "attitude*" OR "Motivat*" OR "influen*" OR "belief*" OR "interest*" OR "hobby*"OR "factor") OR AB ("barrier*" OR "facilitat*" OR "attitude*" OR "Motivat*" OR "influen*" OR "belief*" OR "interest*" OR "hobby*"OR "factor") | **1,210,121**  **27926**  **1238047** |
| **#4** | (DE "exercise*") OR TI （"physical activit*" OR "exercise*" OR "sport*"） OR AB （"physical activit*" OR "exercise*" OR "sport*"） | **327,620**  **7394**  **335014** |
| **#5** | (DE "student*") OR (DE "teacher*")) OR (DE "young adult*") OR (DE "youth") OR TI ("student*" OR junior* OR "undergraduate*" OR "university*" OR "teacher*" OR "adult*" OR "adolescent*" OR "teenager*" OR "young adult*" OR "physical teacher*" OR "adult children*" OR teen* "OR" "young people" OR "youth" OR "girls" OR "boys" OR "young women" OR "young men") OR AB ("student*" OR junior* OR "undergraduate*" OR "university*" OR "teacher*" OR "adult*" OR "adolescent*" OR "teenager*" OR "young adult*" OR "physical teacher*" OR "adult children*" OR teen* "OR" "young people" OR "youth" OR "girls" OR "boys" OR "young women" OR "young men") | **1,241,466**  **29349**  **1270815** |
| **#6** | #1 AND #2 AND #3 AND #4 AND #5 | **142** |

** Searches made through EBSCO-host*

**APA PsycINFO**

| **Searches** | **Search terms (AB, TI, DE 2024)** | **Results** |
| --- | --- | --- |
| **#1** | (DE "school*") OR TI ( "college*" OR "university*" OR "academy*" OR "school*" OR "high school*" OR "physical education*" OR "inclusive education" OR "middle school*" ) OR AB ("college*" OR "university*" OR "academy*" OR "school*" OR "high school*" OR "physical education*" OR "inclusive education" OR "middle school*") | **732,033**  **12047**  **753,793** |
| **#2** | (DE "disabilities or disabled") OR TI ("physical disabilit*" OR "vision disabilit*" OR "childhood onset disabilit*" OR "motor disabilit*" OR "vision impairment" OR "intellectual disabilit*" OR "hearing impairment" OR "hearing disabilit*" OR "multiple disabiliti*" OR "disabilit*" OR "impairment*" OR "disabled person" OR "disabled children" OR "disabled student" OR "extremity disability" OR "upper extremity disability" OR "lower extremity disability" OR "wheelchair" OR "Sports for Persons with "Disabilities") OR AB ("physical disabilit*" OR "vision disabilit*" OR "childhood onset disabilit*" OR "motor disabilit*" OR "vision impairment" OR "intellectual disabilit*" OR "hearing impairment" OR "hearing disabilit*" OR "multiple disabiliti*" OR "disabilit*" OR "impairment*" OR "disabled person" OR "disabled children" OR "disabled student" OR "extremity disability" OR "upper extremity disability" OR "lower extremity disability" OR "wheelchair" OR "Sports for Persons with "Disabilities") | **86,733**  **1551**  **88284** |
| **#3** | (DE "attitude*") OR (DE "Motivat*")) OR (DE "influen*") OR (DE "belief*") OR (DE "interest*") OR TI ("barrier*" OR "facilitat*" OR "attitude*" OR "Motivat*" OR "influen*" OR "belief*" OR "interest*" OR "hobby*"OR "factor") OR AB ("barrier*" OR "facilitat*" OR "attitude*" OR "Motivat*" OR "influen*" OR "belief*" OR "interest*" OR "hobby*"OR "factor") | **1,695,320**  **29739**  **1725059** |
| **#4** | (DE "exercise*") OR TI （"physical activit*" OR "exercise*" OR "sport*"） OR AB （"physical activit*" OR "exercise*" OR "sport*"） | **179,446**  **3340**  **182786** |
| **#5** | (DE "student*") OR (DE "teacher*")) OR (DE "young adult*") OR (DE "youth") OR TI ("student*" OR junior* OR "undergraduate*" OR "university*" OR "teacher*" OR "adult*" OR "adolescent*" OR "teenager*" OR "young adult*" OR "physical teacher*" OR "adult children*" OR teen* "OR" "young people" OR "youth" OR "girls" OR "boys" OR "young women" OR "young men") OR AB ("student*" OR junior* OR "undergraduate*" OR "university*" OR "teacher*" OR "adult*" OR "adolescent*" OR "teenager*" OR "young adult*" OR "physical teacher*" OR "adult children*" OR teen* "OR" "young people" OR "youth" OR "girls" OR "boys" OR "young women" OR "young men") | **1,648,396**  **26455**  **1674851** |
| **#6** | #1 AND #2 AND #3 AND #4 AND #5 | **183** |

** Searches made through EBSCO-host*

Supplementary Data B. Appraisal of studies.

|  | Devine 2013 | Devine  2016 | Chen  2023 | Orr  2018 | Apelmo 2019 | Downs  2021 | Haegele  2017 | Yessick  2019 | Hortigüela-Alcal  2022 | Buckley  2021 | Coates  2023 |
| --- | --- | --- | --- | --- | --- | --- | --- | --- | --- | --- | --- |
| CASP Qualitative Checklist | 8/10 | 9/10 | 10/10 | 10/10 | 9/10 | 10/10 | 10/10 | 10/10 | 10/10 | 9/10 | 10/10 |
| 1.Was there a clear statement of the aims of theresearch? | 🗸 | 🗸 | 🗸 | 🗸 | 🗸 | 🗸 | 🗸 | 🗸 | 🗸 | 🗸 | 🗸 |
| 2.Is a qualitative methodology appropriate? | 🗸 | 🗸 | 🗸 | 🗸 | 🗸 | 🗸 | 🗸 | 🗸 | 🗸 | 🗸 | 🗸 |
| 3.Was the research design appropriate to address | 🗸 | 🗸 | 🗸 | 🗸 | 🗸 | 🗸 | 🗸 | 🗸 | 🗸 | 🗸 | 🗸 |
| 4.Was the recruitment strategy appropriate to the aims of the research? | ? | 🗸 | 🗸 | 🗸 | 🗸 | 🗸 | 🗸 | 🗸 | 🗸 | 🗸 | 🗸 |
| 5.Was the data collected in a way that addressed the research issue? | 🗸 | 🗸 | 🗸 | 🗸 | 🗸 | 🗸 | 🗸 | 🗸 | 🗸 | 🗸 | 🗸 |
| 6. Has the relationship between researcher and participants been adequately considered? | ? | ? | 🗸 | 🗸 | ? | 🗸 | 🗸 | 🗸 | 🗸 | ? | 🗸 |
| 7.Have ethical issues been taken into consideration? | 🗸 | 🗸 | 🗸 | 🗸 | 🗸 | 🗸 | 🗸 | 🗸 | 🗸 | 🗸 | 🗸 |
| 8.Was the data analysis sufficiently rigorous? | 🗸 | 🗸 | 🗸 | 🗸 | 🗸 | 🗸 | 🗸 | 🗸 | 🗸 | 🗸 | 🗸 |
| 9.Is there a clear statement of findings? | 🗸 | 🗸 | 🗸 | 🗸 | 🗸 | 🗸 | 🗸 | 🗸 | 🗸 | 🗸 | 🗸 |
| 10.How valuable is the research? | 🗸 | 🗸 | 🗸 | 🗸 | 🗸 | 🗸 | 🗸 | 🗸 | 🗸 | 🗸 | 🗸 |
|  | Goodwin  2022 | Haegele 2019 | Haegele  2018 | Haegele 2023 | Haegele 2020 | Qi  2017 | Tanure-  Alves  2024 | Wang  2019 | Wilson  2020 | Grenier  2023 | Asbjørnslett  2008 |
| CASP Qualitative Checklist | 10/10 | 10/10 | 10/10 | 10/10 | 10/10 | 10/10 | 8/10 | 10/10 | 9/10 | 10/10 | 10/10 |
| 1. Was there a clear statement of the aims of theresearch? | 🗸 | 🗸 | 🗸 | 🗸 | 🗸 | 🗸 | 🗸 | 🗸 | 🗸 | 🗸 | 🗸 |
| 2.Is a qualitative methodology appropriate? | 🗸 | 🗸 | 🗸 | 🗸 | 🗸 | 🗸 | 🗸 | 🗸 | 🗸 | 🗸 | 🗸 |
| 3.Was the research design appropriate to address | 🗸 | 🗸 | 🗸 | 🗸 | 🗸 | 🗸 | 🗸 | 🗸 | 🗸 | 🗸 | 🗸 |
| 4.Was the recruitment strategy appropriate to the aims of the research? | 🗸 | 🗸 | 🗸 | 🗸 | 🗸 | 🗸 | 🗸 | 🗸 | 🗸 | 🗸 | 🗸 |
| 5.Was the data collected in a way that addressed the research issue? | 🗸 | 🗸 | 🗸 | 🗸 | 🗸 | 🗸 | 🗸 | 🗸 | 🗸 | 🗸 | 🗸 |
| 6. Has the relationship between researcher and participants been adequately considered? | 🗸 | 🗸 | 🗸 | 🗸 | 🗸 | 🗸 | ? | 🗸 | ? | 🗸 | 🗸 |
| 7.Have ethical issues been taken into consideration? | 🗸 | 🗸 | 🗸 | 🗸 | 🗸 | 🗸 | ? | 🗸 | 🗸 | 🗸 | 🗸 |
| 8.Was the data analysis sufficiently rigorous? | 🗸 | 🗸 | 🗸 | 🗸 | 🗸 | 🗸 | 🗸 | 🗸 | 🗸 | 🗸 | 🗸 |
| 9.Is there a clear statement of findings? | 🗸 | 🗸 | 🗸 | 🗸 | 🗸 | 🗸 | 🗸 | 🗸 | 🗸 | 🗸 | 🗸 |
| 10.How valuable is the research? | 🗸 | 🗸 | 🗸 | 🗸 | 🗸 | 🗸 | 🗸 | 🗸 | 🗸 | 🗸 | 🗸 |

Note:H=high; ✓=YES;**×**=NO;?= Can't tell; CASP= Critical Appraisal Skills Programme

Supplementary Data C. Summary of study characteristics (n= 22).

| **First Author**  **(Year)** | **Subject Category** | **Qualitative Method**  **and Theoretical**  **Perspectives^a^** | **Data Collection and Analysis** | **Sample**  **size^b^** | **Age and**  **Gender^b^** | **Disability Type** | **Purpose of Research and Phenomena of Interest** |
| --- | --- | --- | --- | --- | --- | --- | --- |
| #1  Devine 2013 | SWD(U) | **Method:**  Classical Grounded Theory  **Theory:**  Theory of Involvement | **Data:**  In-depth interviews  **Analysis:**  Grounded theory constant  comparative method | N=16 | **Gender:**  7M/9F  **Aged:**  18-24 | Paraplegia, Cerebral Palsy,  Spinal Muscular Atrophy,  Macular Degeneration,  Blindness, Spina Bifida,  Double AK Amputation | The article explores the perceptions of college SWD after-school PA on campus, examining facilitating and hindering factors. The results expand the context and target au-dience of this study, providing a unique perspective in a higher educationsetting. |
| #2  Devine 2016 | SWD(U) | **Method:**  Classical Grounded Theory  **Theory:**  Not specified | **Data:**  In-depth interviews,  open-ended interviews  **Analysis:**  Grounded theory constant  comparative method | N=16 | **Gender:**  7M/9F  **Aged:**  18-24 | Cerebral Palsy, Paraplegia,  Macular Degeneration,  Multiple Sclerosis,  Spina Bifida,  Double Above-Knee Amp-utation, Blindness,  Spinal Muscular Atrophy | The article explores the experiences of college SWD in leisure-time PA, focusing on facilitating and hindering factors. It expands the study's context and subjects, offering a unique perspective in higher education settings. |
| #3  Chen 2023 | SWD(U) | **Method:**  Phenomenopgraphic  **Theory:**  Social-ecological model | **Data:**  In-depth interviews  **Analysis:**  Thematic analysis | N=8 | **Gender:**  6M/2F  **Aged:**  19-28 | Intellectual disabilities | The article uses qualitative methods to study the views of students with intellectual disabilities during their transition from secondary to higher education. It analyzes intrinsic factors, peer support, and experiences at university recreational centers based on a s Social-ecological framework. |
| #4  Orr  2018 | SWD(S) | **Method:**  Deductive thematic approach.  **Theory:**  Self-determination theory | **Data:**  Semi-structured interviews,  Relational mapping, Field notes  **Analysis:**  Thematic analysis | N=8 | **Gender:**  5M/3F  **Aged:**  13-18 | Cerebral palsy,  Hip growth plate damage,  Developmental coordination disorder (high-functioning autism spectrum disorder),  Arthrogryposis,  Double below-the-knee amputation | The article explores the sports experiences of youth SWD and the role of peers. The study focuses on interactions with classmates and PE teachers, highlighting dynamic peer relationships in the sports context. |
| #5  Apelmo 2019 | SWD(S) | **Method:**  Phenomenology  **Theory:**  Not specified | **Data:**  Semi-structured interviews  **Analysis:**  Hermeneutic approach | N=13 | **Gender:**  3M/10F  **Aged:**  F:15–28  M: 10–15 | Not specified | The article explores how young sporting women with physical impairments experiencephysical education from a gender perspective, andthe strategies they use to manage situations. While boys and a male coach were included for comparison, we are interested in whether gender norms act as facilitating or hindering factors, and the experiences of exclusion and special treatment faced by female participants in mainstream schools. |
| #6  Downs 2021 | SWD(Y)  and Doctors | **Method:**  Interpretive description  **Theory:**  Not specified | **Data:**  Focus groups  **Analysis:**  Inductive thematic | N=6  N=7(D) | **Gender:**  4M/2F;  **Aged:**  11-22;  N/R(D) | Childhood-onset physical disa-bility | The article investigates the current PA experiences of youth SWD and their expectations as they transition to adult services. While focusing on rehabilitation centers, the study provides valuable insights into challenges faced in mainstream schools and clinicians' perspectives on school accessibility and teacher training. |
| #7  Haegele 2017 | SWD(A) | **Method:**  Interpretative pheno-menological analysis  **Theory:**  Not specified | **Data:**  Semistructured audiotaped  telephone interviews,  Reflective field notes  **Analysis:**  Interpretative analysis | N=16 | **Gender:**  6M/10F  **Aged:**  21-48 | Visual Impairment | The article examines the experiences of adults with visual impairments during school-based integrated PE. Despite potential limitations of retrospective studies, the findings on factors influencing participation are valuable for understanding the impact on adult. |
| #8  Yessick 2019 | SWD(A) | **Method:**  Exploratory  **Theory:**  Not specified | **Data:**  Semi-structured,  Audiotaped telephone inter-views,  Reflective field notes  **Analysis:**  Inductive analysis | N=16 | **Gender:**  7M/9F  **Aged:**  21-48  Mv=31.88 | Visual Impairment | The article explores the impact of PE experiences on current PA participation among adults with SWD. The study focuses on theireducational experiences in integrated settings and how teachers' attitudes, accommodations, and opportunities influenced their participation. |
| #9  Hortigüela-Alcal 2022 | PE teachers  and  SWD(U)  and  SWD(P) | **Method:**  Retrospective design  **Theory:**  Social identity  Social desirability | **Data:**  Interviews,  Narrative journals,  Discussion groups  **Analysis:**  Inductive coding,  Content analysis | N=3(S)  N=3(P)  N=3(T) | **Gender:**  1M/2F  2M/1F  2M/1F  **Aged:**  20-21(S) | Arm amputation,  Hip dysmetry,  Tourette’s syndrome | The article analyzes the motivations of future PE teachers with motor disabilities. While the focus is on career motivations, we are interested in the participants' sports experiences as noted by themselves, their parents, and teachers, which influenced their career choice. |
| #10  Buckley 2021 | SWD(S) | **Method:**  Phenomenopgraphic,  Retrospective design  **Theory:**  Not specified | **Data:**  Semi-structured,  Audiotaped,telephone/video interviews,  Reflective interview notes  **Analysis:**  Thematic analysis | N=4 | **Gender:**  4F  **Aged:**  23-28 | Visual Impairment | The article aims to understand how individuals identifying as female, athletes, and having visual impairments experience PE and sports. Despite reporting negative school experiences, their multifaceted identities providevaluable insights for ourstudy. |
| #11  Coates  2023 | SWD(S)  and  SWD(P) | **Method:**  Existential phenome-nology  **Theory:**  Phenomenology,  (focusing on life histories and reflective experiences) | **Data:**  Semi-structured interviews  **Analysis:**  Reflexive thematic analysis | N=10(S)  N=11(P) | **Gender:**  7M/3F(S)  3M/8F(P)  **Aged:**  12-25(S)  M V=16.6 | Not specified | The article reviews parents' experiences supporting their disabled children in parasport, focusing on their dissatisfaction with mainstream and school sports and their children's participation experiences. |
| #12  Goodwin2022 | SWD(A) | **Method:**  Interpretative pheno-menological analysis  **Theory:**  Relational ethics | **Data:**  Semi-structured interviews, In-depth interviews.  **Analysis:**  Thematic analysis | N=5 | **Gender:**  2M/3F  **Aged:**  18-30  Mv=23 | Not specified | The article examines the impact of paraeducators on SWD's participation in PA during elementary, middle, and high school. We focus on the middle and high school stages and the facilitating or hindering factors by paraeducators in educational practice. |
| #13  Haegele 2019 | SWD(Y) | **Method:**  phenomenological research approach  **Theory:**  Not specified | **Data:**  Semistructured face-to-face interviews,  Reflective field notes,  Observation notes  **Analysis:**  Thematic analysis | N=4 | **Gender:**  3M/1F  **Aged:**  11-16 | Visual Impairment | The article explores the PE experiences, attitudes, values, and feelings of Alaskan youths with visual impairments in integrated public schools. |
| #14  Haegele 2018 | SWD(A) | **Method:**  Interpretative phenomenological analysis,  Retrospective design  **Theory:**  Intersectionality | **Data:**  Semistructured,  Audiotaped, Telephone int-erview,  Reflective field note  **Analysis:**  Thematic analysis | N=6 | **Gender:**  6M  **Aged:**  18-33 | Visual Impairment | The article uses an intersectional approach to examine visually impaired adult males' experiences in integrated PE. We are interested in how multiple identities and nonnormative bodies affect their PE experiences. |
| #15  Haegele 2023 | SWD(Y) | **Method:**  Experiential qualitat-ive  **Theory:**  Relativist ontology | **Data:**  Semi-structured interviews  **Analysis:**  Reflexive thematic analysis | N=18 | **Gender:**  11M/7F  **Aged:**  12-15 | Visual Impairment | The article explores visually impaired youths' views on teacher initiated activity modifications during integrated PE. The findings support the necessity of changing teaching strategies and behaviors. |
| #16  Haegele 2020 | SWD(A) | **Method:**  Interpretative phenomenological analysis,  Retrospective design  **Theory**  Relativist ontology,  Subjective epistemol-ogy | **Data:**  Semi-structured audiotaped,  Telephone interviews,  Reflective interview notes  **Analysis:**  Thematic analysis | N=11 | **Gender:**  4M/7F  **Aged:**  20-35 | Visual Impairment | The article describes the fitness testing experiences of visually impaired adults in integrated PE, highlighting the context of mainstream schools and potential impacts on educational practices. |
| #17  Qi  2017 | PE teachers | **Method:**  Not specified  **Theory:**  Lev Vygotsky’s  social constructivism | **Data:**  Semi-structured interviews,  **Analysis:**  Content analysis,  Analytical framework | N=8 | **Gender:**  3M/5F  **Aged:**  28-37  Mv=31.88 | Autism spectrum disorders,  Attention deficit hyperactivity disorder,  Intellectual Disability,  Visual impairments,  Special learning difficulty | The article examines the perceptions of Hong Kong secondary PE teachers regarding the inclusion of students with disabilities in general PE programs. |
| #18  Tanure Alves 2024 | PE teachers | **Method:**  Exploratory  **Theory:**  Not specified | **Data:**  Focus group interviews,  Reflective journal with fiel-d note  **Analysis:**  Content analysis | N=3 | **Gender:**  N/R  **Aged:**  N/R | Not specified | The article aims to understand the ableism facing disabled students in PE classes. We are interested in the difficulties encountered in including SWD in PE. |
| #19  Wang 2019 | SWD(Y) | **Method:**  Not specified  **Theory:**  Social relational  model | **Data:**  Semistructured interviews  **Analysis:**  Content analysis | N=20 | **Gender:**  13M/7F  **Aged:**  12-16  Mv=13 | Physical impairments,  Autism spectrum disorders,  Intellectual disabilities,  Visual impairments,  Health impairments,  Hearing impairment | The article explores the perceptions of SWD on inclusion in PE in the Chinese educational context, using the social relational model of disability. We are interested in the identified personal, physical, and social context factors. |
| #20  Wilson 2020 | PE teachers  and  APE teachers | **Method:**  Not specified  **Theory:**  Occupational socialisa-tion | **Data:**  Open-ended response item  **Analysis:**  Thematic analysis | N=30 T  N=48 A | **Gender:**  27M/51F  **Aged:**  Mv=42.8 T  Mv=46.2 A | Not specified | The article examines PE and adapted PE teachers’ perspectives on inclusive practices in integrated PE. We are interested in how educators facilitate PA for SWD and the challenges they face. |
| #21  Grenier 2023 | SWD(P)  and  APE  Teachers  and  Paraprofessional | **Method:**  Not specified  **Theory:**  Universal Design for Learning framework | **Data:**  Focus group interviews,  Reflexive note  **Analysis:**  Inductive coding | N=11 | **Gender:**  N/R  **Aged:**  34-63  Mv=45.45 | Visual impairments and additional disability | The article determines the training needs of teachers and support staff for children with visual impairments and additional disabilities through parents' and educators' voices. We areinterested in the insights provided by the Universal Design for Learning framework, which explores barriers to participation in PE from multiple stakeholders. |
| #22  Asbjørnslett  2008 | SWD(Y) | **Method:**  Not specified  **Theory:**  Not specified | **Data:**  Focus-group interviews,  Individual interviews  **Analysis:**  Thematic analysis | N=14 | **Gender:**  8M/6F  **Aged:**  13-18 | Physical disability | The article investigates the experiences of SWD in mainstream school education, with a specific focus on their PE experiences. |

Note: Mv = Mean; M = Male; F = Female; N/R = Not Reported. PA=physical activity; PE= physical education; APE= Adapted physical education; SWD= Students with disabilities; SWD(U)=Students with Disabilities as College Students; SWD(P) = parent of student with a disability; SWD(S)= students with disabilities who identify as athletes; SWD(Y) = Students with Disabilities in Young Adults; SWD(A)= Adults with disabilities recalling their time as students with disabilities.

^a^ In this table, methodology refers to the specific qualitative research method adopted for each study (e.g., phenomenological, grounded theory, ethnography, etc.).

^b^. The **s**uffix“S”in these two columns represents disabled students, the suffix“T” represents general physical education teachers, the suffix“A”represents adapted physical education teachers, the suffix“P”represents the parents of disabled students, and the suffix“D”represents doctors.

Supplementary Data D. Theme Extraction Table and Path.

| **Stakeholder** | **Thematic** | | | | | **Study** |
| --- | --- | --- | --- | --- | --- | --- |
| SWD | Special factor | | | Biological problem | | Devine, 2013;  Devine, 2016;  Chen et al., 2023;  Orr et al., 2018;  Haegele & Zhu, 2017;  Buckley et al., 2021;  Tanure Alves et al., 2024;  Wang,2019;  Wilson et al., 2020; |
|  |  |  |  | Sports fundamentals | |  |
|  |  |  |  | Individual Characteristics | |  |
|  |  |  |  | Own PA attitude | |  |
|  |  |  |  | Targets obtained through PA | |  |
| School  administration | First  phase | School attitudes and policies | | | | Devine, 2016;  Chen et al., 2023;  Downs et al., 2021;  Coates & Howe, 2023;  Qi et al., 2017;  Wilson et al., 2020;  Grenier et al., 2023 |
|  |  | School support | | Teaching resource | |  |
|  |  |  |  | Human resources | |  |
|  |  |  |  | Qualified teachers | |  |
| Teaching &  Administrative staff | Second  phase | Expertise capacity | | | | Haegele et al., 2023;  Qi et al., 2017;  Wilson et al., 2020; |
|  |  | Application capacity | | | |  |
|  |  | Collaboration-Teaching support environment | | | |  |
| PE & SWD & Peer | Third  phase | Competence need | | | Physical Barriers | Devine, 2013;  Devine, 2016;  Orr et al., 2018;  Downs et al., 2021;  Yessick & Haegele, 2019;  Haegele & Buckley, 2019;  Haegele & Kirk, 2018;  Haegele et al., 2023;  Haegele et al., 2020;  Qi et al., 2017;  Tanure Alves et al., 2024;  Wang, 2019;  Wilson et al., 2020;  Asbjørnslett & Hemmingsson, 2008; |
|  |  |  |  |  | Layout and Design of Sports Facilities |  |
|  |  |  |  |  | Flexible Teaching Arrangements  Effective Instructional Guidance |  |
|  |  |  |  |  | Modified Teaching Content |  |
|  |  | Relationship need | | | Teacher-Student Connectedness | Devine, 2013;  Devine, 2016;  Apelmo, 2019;  Downs et al., 2021;  Haegele & Zhu, 2017;  Yessick & Haegele, 2019;  Buckley et al., 2021;  Coates & Howe, 2023;  Goodwin et al., 2022;  Haegele & Buckley, 2019;  Haegele & Kirk, 2018;  Haegele et al., 2023;  Haegele et al., 2020;  Qi et al., 2017;  Wang, 2019; |
|  |  |  |  |  | Three Forms of Peer Interaction |  |
|  |  |  |  |  | Special Attention |  |
|  |  | Autonomous need | | | Forced exclusion | Devine, 2013;  Devine, 2016;  Chen et al., 2023;  Apelmo, 2019;  Haegele & Zhu, 2017;  Hortigüela-Alcalá et al., 2022;  Coates & Howe, 2023;  Goodwin et al., 2022;  Haegele & Buckley, 2019;  Haegele & Kirk, 2018;  Haegele et al., 2020;  Wang, 2019;  Grenier et al., 2023;  Asbjørnslett & Hemmingsson, 2008; |
|  |  |  |  |  | Activity diversity |  |
|  |  |  |  |  | Opportunities for self-determination |  |
|  | | | | | | |
| **Stakeholder interaction** | | | **Dynamic relational path** | | | |
| School Administration  Teaching and Administrative staff | | | School support → Expertise capacity;  School support → Application capacity;  School support → Collaboration  Collaboration → Teaching support environment | | | |
| Teaching and Administrative staff  PE & SWD & Peer | | | Expertise capacity → Competence need  Expertise capacity → Relationship need  Expertise capacity → Autonomous need  Expertise capacity → Teaching support environment → Competence need  Expertise capacity → Teaching support environment → Relationship need  Expertise capacity → Teaching support environment → Autonomous need  Competence need ↔ Relationship need ↔ Autonomous need | | | |
| SWD  School Administration  Teaching and Administrative staff  PE & SWD & Peer | | | “Special factor ”↔“First phase”↔“Second phase”↔“Third phase” | | | |

Note:SWD: Students with disabilities;PE: Physical Education Teacher (*Uniform designation for staff in the comprehensive school context in this study*);

Supplementary Data E.A summary table of barriers and facilitators affecting SWD students in an inclusive school setting based on the 22 articles incorporated in this review

| lack of administrative support | appropriate learning environment | 【curriculum is not properly arranged 】  【resources are insufficient】  【 special classes are low priority】 |
| --- | --- | --- |
|  | Teaching equipment support | 【Special teaching equipment support】  【No place to store the equipment】  【Expensive equipment】  【 No sponsorship for purchase/ Small budget】 |
|  | personnel support | 【Physical education teachers take on too many roles】 |
|  | attention of the school management | 【 School's policy direction is inclusive】  【Inclusive slogan】  【Managers should attach importance to physical exercise and focus on increasing the correlation between physical exercise and academic performance】 |
| Establish ground rules | Paraprofessionals accommodate physical  education teachers | 【Paraprofessionals treat physical education as a break】  【No action was taken after communication with management】  【Paraprofessionals should be properly trained】 |
|  | Clear guidance and assessment | 【School provides more indicators/ assessment criteria】  【Policies can provide guidance or goals for how we educate students with disabilities】  【Current evaluation only depends on the adjustment of teachers themselves】 |
| Training and Seminars | Professionalism of training seminars and the richness of sharing | 【 professional training provided by schools is very inadequate】  【Lectures and consultations are unprofessional】  【Educational psychologists have not spent a great deal of practice discussing students with disabilities in educational contexts】  【The seminar experience gave me a basic understanding of how to teach physical education to students and prepared me in advance】 |
| School atmosphere | Supportive atmosphere | 【The support of my classmates and teachers, the whole school supported me】 |
| Teacher's attitude | Whether to treat equally | 【Treat me like a child, not a blind man】  **【**The PE teacher spoke to us in a somewhat abnormal tone, as if he were talking to abnormal people】 |
|  | Whether you care about | 【 Teacher doesn't care. She just thinks I'm in the way】  【Teachers only care about their football teammates】  【Teachers care about us】  【 teacher cared about my feelings and safety】 |
|  | Low expectations/exclusions from teachers | 【Do alternative activities】  【Participate in parallel activities away from classmates】  【Do something unrelated to physical activity】  【No chance to participate in sports】  【no chance to experience different activities】  【Being treated differently.】  【The teacher didn't think I could do it】  【There are no alternative activities】  【Unable to integrate into physical activities】  【The teacher didn't trust my own method of exercise】  【Give me the win in the classmate competition】  【The teacher thought it was too difficult to teach me to dribble】  【Unable to participate in the competitive culture of mainstream sports classes】  【The teacher never said to me, "I hope you do better."】  【Teachers want to see a high level of competition】  【All I have to do is show up, participate and get my grade】  【Some behaviors of disabled students are considered dangerous】  【 Participate in most activities, but you are not allowed to participate in team activities 】  【Strenuous exercise is not allowed】  【security concerns】 |
|  | Completely ignore students’disabilities | 【Ignoring students' actual physical limitations】  【Students feel pain and illness during exercise】  【Students with less visible disabilities - are stressed beyond their personal physical limits】  【Tell teachers that needs are being ignored】 |
| Professional knowledge ability | | 【Teachers don't really have the knowledge and framework to make these decisions】  【lack of abilities to deal with the disciplinary problems】  【Teachers don't have adaptive knowledge】  【Teachers lack preparation for students with disabilities】  【Understand the different barriers and how they affect students' abilities】  【Focus on the sensory system for education】 |
| "False integration" | | 【Just get credit.】  【Teachers don't really work hard and make sure we do well or learn something】  【feel like an obligation】 |
| Support for teachers | A supportive environment for teaching and learning among staff | 【Communicate with the school's social worker to get information and discuss solutions】  【Find peers who are taking the course and ask for some advice or determine if we can work together】  【Teachers of all kinds hold group meetings together】  【Develop collaborative relationships with other school staff】  【Develop collaborative relationships with other school staff】  【Early assessment of APE teachers】  【APE teachers train PE teachers to keep their knowledge up to date】 |
|  | Management/school policy support for teachers | 【The school did not provide teachers with documentation related to students with disabilities】  【Insufficient support for detailed information for students with disabilities】  【Physical education teachers have no say in school】  【Physical education teachers cannot be arranged in ordinary teaching Settings】 |
| Special characteristics of PE class | Expose oneself to disability | 【Physical education exposes their shortcomings, so that the psychological feeling more uneasy】 |
| Teaching arrangement | Activity intensity in class | 【Can not participate in high intensity and high-density activities】 |
|  | Availability of class location and environment/venue | 【The grass is not accessible to wheelchairs】  【There is a railing between the door and the door, and it is impossible to enter the gym with a sports chair】  【Wheelchair accessibility】  【There was no shade in the classroom】  【There are many stairs on the way to class and no elevator】 |
|  | Availability of teaching tools | 【The color of the teaching equipment】  【The height of the teaching equipment】  【The volume of the teaching equipment】  【Materials of teaching tools】  【Modified teaching equipment】 |
|  | Teaching Scale (Number of students) | 【The class size is so large that it is difficult to modify all the activities for the students】 |
|  | Differences in disability among classes | 【Teachers cannot always pay attention to students with different types of disabilities】  【Teachers do not have the confidence to face students with disabilities】  【Although teachers are prepared in advance, they do not have a strong professional capacity to teach different types of students with disabilities】 |
|  | Arrangement of the class time | 【Lack of a curriculum suitable for students with disabilities】 |
| Teaching methods | Teacher and student interaction/communication | 【Students take the initiative to communicate their own situation and needs】（+）  【timely communication】（+）  【The teacher asks for and accepts the students' suggestions】（+）  【Independence is gained in making decisions and solving interdependent problems】（+）  【A one-way communication channel】  【Repeated requests for changes are tiring and patience is limited】 |
|  | Teachers' support for students | 【It takes more time to explain the skill acquisition】  【Teachers offer individual activities to exercise motor skills】  【APE teacher's help/Support staff help】  【Physical therapists help students modify activities】  【Arrange a friend for a disabled student】  【APE's one-on-one instruction to students】  【Common teaching of physical education teachers and APE teachers】  【Individual skills training activities outside of class】  【The need for additional guidance is not being met】  【The teacher cannot guarantee the safety of the students】 |
|  | Explanation/Pace of lessons | 【tactile modeling and physical guidance】  【The teacher's explanation confused the students】  【The practice time in class is too short】  【Too fast class pace】 |
|  | Classroom flexibility | 【Teacher believes that the disabled students are very interested in the dynamic use of games and activities】  【 lack of flexibility and unwillingness to change learning styles】 |
|  | **special attention** | 【Special care has attracted special attention】  【Inappropriate behavior by teachers】  【Treating participants in a manner beyond reasonable bounds】 |
| teaching style | Encouraging style | - |
|  | Casual style | 【The teacher won't help correct my movements】  【Teachers are not supportive/helpful】 |
|  | Suppressive style | 【Difficult teaching task】  【teacher told me to give it up】 |
| Content of courses | Unmodified team game rules | - |
|  | Unmodified/complex teaching content | 【Complex motor skills】 |
|  | Revised teaching content | 【Teachers create more opportunities to participate in PA】  【Professional involvement in teaching modification】  【Allow ordinary students to experience inclusive teaching activities】  【The teacher put himself in student place】  【Teachers' motivation to make changes】  【A game was added to the class to get disabled students involved】  【Get the students involved】  【"Band-aid type" modification】（Modifications are understood as only minimal improvements to enable them to participate, essentially band-aids for activities or courses that do not take their needs into account）  【Brief game modification】 |
|  | Paralympic sports | 【The Paralympics are just an experience】  【Only one Paralympic physical activitie class a year】 |
|  | Fun sports games | - |
| Teaching atmosphere | Inclusive atmosphere | 【The welcoming atmosphere of the class and collaborative learning in the classroom】 |
| cooperation | Paraprofessionals accommodate physical education teachers | 【APE teachers prepare well in advance to communicate the main points of each lesson to support staff】 |
| No responsibility, no action | | 【The teaching assistant exercises his right of exclusion through inaction】 |
| Social interactions lack transparency | Overprotection | 【paraeducator benevolent care that continued to stand between student and classmates.】  【paraeducator takes participants out of gym class without counseling】 |
| Negative interaction | Bullying/teasing | 【Verbal teasing】  【physical violence】  【Silly nickname】  【When you improve your ability, you gain respect】 |
|  | Peer exclusion | 【Be ignored by peers】  【Classmates prevented me from participating in sports activities】  【Was prevented from participating in competitive events】  【Being prevented from participating in team activities】  【Rarely talk to me】  【No one wants to partner with a disabled student】 |
|  | Negative emotional experience of team activities | - |
|  | "last choice." | 【No students team up with disabled students unless they have to】  【Children are ruthless】 |
| one's abilities are strong | A sense of mastery of ability | 【Students try their best to complete intensive exercises】  【Students take pride in showing their ability】 |
| own negative feelings. | Low self-efficacy | 【be not qualified】.  【fear of failure】  【Lack of ability, dare not participate in team activities】  【Feeling inferior to other students】 |
|  | Embarrassed | 【difference is obvious】  【The embarrassment of not being able to complete the move】 |
| Disability itself | Physical disorder | 【It's hard to jump in a wheelchair. 】  【Doctors advise students with heart disease not to exercise too intensely】  【Has gastroesophageal reflux disease】  【mental retardation】 |
| Attitude towards sports | Disinterest/negative perception | 【Previous bad team sports experience】  【There's nothing one wants to learn in physical education class.】  【Independent choice】  【Negative view of sports】  【It doesn't matter if you don't exercise in PE class. PE class is more like a social platform】  【students are not interested】 |
| Personal character | Proactively ask for help and modifications | 【Extroverted personality actively asks for help】 |
